# Transcutaneous posterior tibial nerve stimulation in children and adolescents with functional constipation: A protocol for an interventional study

**DOI:** 10.1097/MD.0000000000017755

**Published:** 2019-11-11

**Authors:** Rebeca Mayara Padilha Rego, Nilton Carlos Machado, Mary de Assis Carvalho, Johann Souza Graffunder, Erika Veruska Paiva Ortolan, Pedro Luiz Toledo de Arruda Lourenção

**Affiliations:** a UNESP - São Paulo State University, Botucatu Medical School, Botucatu; b UNESP - São Paulo State University, Botucatu Medical School, Department of Pediatrics, Pediatric Gastroenterology, Hepatology and Nutrition Division, Botucatu; c UNESP - São Paulo State University, Botucatu Medical School, Department of Surgery and Orthopedics, Pediatric Surgery Division, Botucatu, SP, Brazil.

**Keywords:** child, intestinal constipation, neurostimulation

## Abstract

**Introduction:**

A vast majority of children with functional constipation respond to the standard medical treatment. However, a subset of patients may present with an unsatisfactory response and only minor improvement of symptoms. Transcutaneous posterior tibial nerve stimulation (PTNS) involves electrical stimulation of the posterior tibial nerve at the level of the ankle, transcutaneously through electrodes fixated on the overlying skin. Stimulation of the tibial nerve can modulate urinary and defecatory function through the stimulation of sacral nerves. Thus, transcutaneous PTNS can be considered a very promising, noninvasive, and safe method to be used in the pediatric age group. However, there is still no published study that has investigated its use in children for the treatment of intestinal constipation. This is a single-center, prospective, longitudinal, and interventional study designed to assess the applicability and clinical outcomes of transcutaneous PTNS in children with functional intestinal constipation. Children will be submitted to daily sessions of transcutaneous PTNS for a period of 4 weeks. All children will also be invited to participate in semistructured interviews, 1 in each of the 3 assessments: 1 week before the start of the intervention; immediately after the 4 weeks of intervention; and 4 weeks after the end of the intervention period. In these interviews, the aspects related to bowel habits and quality of life will be assessed. This project aims to evaluate the clinical outcomes of transcutaneous PTNS in children with functional intestinal constipation and the applicability of this kind of treatment.

**Conclusions:**

This protocol intended to demonstrate the efficacy of this promising method to increase the number of bowel movements and the stool consistency, to reduce the number of episodes of retentive fecal incontinence, and to indirectly improve the overall quality of life.

## Abstract 2

Supplemental Digital Content is available in the text

## 1 Introduction

Functional defecation disorders include functional constipation (FC) and functional nonretentive fecal incontinence (FNRFI). FC represents a frequent reason for healthcare consultations. In a systematic review and meta-analysis that include studies using the Rome III criteria, the prevalence of FC ranged from 0.5% to 32%, with a pooled prevalence of 9.5% (95% confidence interval [CI] 7.5–12.1). For FNRFI, the prevalence ranged from 0.0% to 1.8%, with a pooled prevalence of 0.4% (95% CI 0.2–0.7).^[1]^ Additionally, the prevalence of FC was 8.6% in boys compared with 8.9% in girls (odds ratio 0.99, 95% CI 0.9–1.4). Around 3% of general pediatric outpatient visits and approximately 25% of pediatric gastroenterology consultations are related to a perceived defecation disorder, a significant proportion of which is constipation, with less than 5% of these children have an underlying organic cause.^[2,3]^ In children with FC, painful defecation, fecal incontinence, and abdominal pain may trigger emotional burdens.^[4–6]^ Also, FC is often associated with a significant impact on quality of life.^[7]^

The recommendations for diagnosis are based on history and physical examination and the Rome criteria should be used to define FC. The Rome IV criteria for children and adolescents^[8]^ and neonate and toddler,^[9]^ is a symptom-based consensus process constructed by expert clinicians centered on a literature review.^[10]^ According to the new Rome IV criteria for FC, a distinction from Rome III criteria is made for young children between those who are toilet trained and those who are not. Furthermore, the time criterion has changed, and patients now need to fulfill the criteria for at least 1 month instead of 2 months.^[11]^

In 2014, the European Society for Pediatric Gastroenterology, Hepatology, and Nutrition and the North American Society for Pediatric Gastroenterology, Hepatology, and Nutrition published a guideline that provides evidence-based recommendations for the evaluation, treatment, and follow-up of children with FC.^[3]^ The well-founded point for the treatment of FC consists of education of the child and parents, behavioral modifications, and laxative therapy.^[3,12]^ Therefore, conventional medical management of FC consists of pharmacological and nonpharmacological interventions. The pharmacological treatment of FC with laxatives involves 3 steps; disimpaction, maintenance treatment, and weaning. Nonpharmacological interventions involve education, demystification, explanation, guidance for toilet training, scheduled toilet sits, establishing a reward system, and keeping a defecation diary.^[3,13,14]^

In tertiary care centers, approximately 50% of children with FC managed with conventional treatment will be recover and taken off laxatives within 6 to 12 months, but the others, the symptoms can persist into adulthood despite intensive laxative treatment.^[15]^ Thus, a subset of patients may present with an unsatisfactory response and only minor improvement of symptoms. These children experience severe and long-lasting symptoms with recurrent fecal impactions, clinically characterized by extended intervals between defecation episodes, the passage of a large amount of stools once every 7 to 30 days and fecal incontinence that respond poorly to conventional therapy. Accordingly, when symptoms are irresponsive to optimal conventional treatment for at least 3 months, these children are considered to have intractable FC.^[3,16]^

Unfortunately, treatment options for children with constipation refractory to conventional treatment are limited. The most recent guidelines from the European and North American Pediatric Gastroenterology societies on the evaluation and treatment of FC in children recommend consideration of anal sphincter botulinum toxin injection, transanal irrigation, antegrade continence enemas, sacral nerve stimulation, and partial or total colonic resection for treatment of intractable constipation.^[3,10,17]^

Thus, neuromodulation is one of the treatment modalities that can bring benefits in patients with FC. Transcutaneous electrical stimulation (TES) is a nonpharmacological approach for children with constipation, that facilitate bowel motility, by modulating the nerves of the large bowel.^[18]^ In a systematic review, the effectiveness and safety of TES were evaluated when employed to improve constipation in children.^[19]^ The authors conclude that there are no firm conclusions regarding the efficacy of TES in children with FC, and recommended that randomized controlled trials assessing TES for the management of childhood constipation should be conducted. The future trials should include documentation of methodology and patient outcomes, such as the number of patients with improved clinical symptoms and quality of life.

On the other hand, a subtype of TES termed posterior tibial nerve stimulation (PTNS) involves electrical stimulation at the level of the ankle, delivered either percutaneously by needle punctures or transcutaneous through electrodes fixated on the overlying skin.^[20]^ PTNS transcutaneous can be considered a minimally invasive, and accessible technique with the potential to improve constipation in the pediatric population.^[20,21]^ Stimulation of the tibial nerve can modulate urinary and defecatory function through the stimulation of sacral nerves, similar to those exercised by sacral nervous stimulation (SNS). On the other hand, PTNS has the advantage of not requiring surgical procedures for the implantation of electrodes as in SNS.^[20–22]^ Multiple studies have shown that PTNS is useful for improvement of fecal incontinence in adults and treatment of several urinary dysfunctions in childhood.^[21,23,24]^

In children, only 1 study investigated the applicability of transcutaneous PTNS in a group of 8 children with fecal incontinence associated with a variety of underlying causes, including anorectal malformation, neurological conditions, and Hirschsprung disease.^[21]^ The authors found improvement of fecal incontinence after 6 months in 7 children, and 5 had a resolution of fecal incontinence. Two patients noted the recurrence of fecal incontinence with discontinuation of PTNS treatment.

Considering that there is still a need for better and more effective treatments of childhood FC and that neuromodulation has been incorporated in clinical practice for the treatment of a large number of health problems, ranging from nausea and vomiting to bowel and urinary disorders, and highlighting the potential benefits of this kind of therapy in children with FC who present symptoms refractory to clinical treatment, we decided to assess the applicability and clinical outcomes of transcutaneous PTNS in children with FC.

## 2 Methods

### 2.1 Study design and setting

This is a single-center, interventional, prospective, and longitudinal study design to assess the applicability and clinical outcomes of transcutaneous PTNS in children with FC. This study will be conducted at the Botucatu Medical School, São Paulo State University (UNESP), São Paulo, Brazil. The data collection period will be 12 months long, and started in April 2019.

Children will be submitted to daily sessions of transcutaneous PTNS for a period of 4 weeks. All children will also be invited to participate in semi-structured interviews, 1 in each of the 3 assessments: 1 week before the start of the intervention; immediately after the 4 weeks of intervention; and 4 weeks after the end of the intervention period. In these interviews, the aspects related to bowel habits and quality of life will be assessed.

### 2.2 Ethics approval and consent to participate

This study will be conducted following the principles of the Declaration of Helsinki, ISO14155, Data Protection Act, and the Guidelines for Good Clinical Practice. The research ethics committee (REC) of the Botucatu Medical School, UNESP, São Paulo, Brazil, has approved this study, which was registered under number 80013017.0.0000.5411 (see REC, Supplemental Digital Content 1). The patients and their guardians were previously informed of the purpose of the research, and each signed an informed consent form (ICF) (see ICF, Supplemental Digital Content 2). Patients aged 11 to 18 years signed the respective specific consent form (SCF) (see SCF, Supplemental Digital Content 3). All data will be sent to the REC at the end of the study. The subjects may leave this study at any point in time without any limitations.

The Brazilian Registry of Clinical Trials (Rebec) identifier for this study is RBR-344jq8, obtained on March 13, 2018 (UTN number: U1111-1207-5487), and it is available at http://www.ensaiosclinicos.gov.br.

### 2.3 Patient selection and recruitment

The estimated sample size is 28 patients, calculated for a paired *t* test (before and after intervention), based on an estimated mean increase of 7 defecations per month (according to data from previous PTNS studies^[25,26]^), an estimated standard deviation of changes = 12, a 2-tailed *α* = 0.05 and a test power *β* = 0.85. The sample will be comprised of children selected from the Pediatric Gastroenterology Outpatient and Pediatric Surgery Outpatient of the Clinics Hospital of the Botucatu Medical School -UNESP. The patients will be invited to attend an initial consultation to inform them about the study methods and apply the eligibility criteria.

### 2.4 Eligibility criteria

The inclusion and exclusion criteria are presented in Table 1.

**Table 1:**
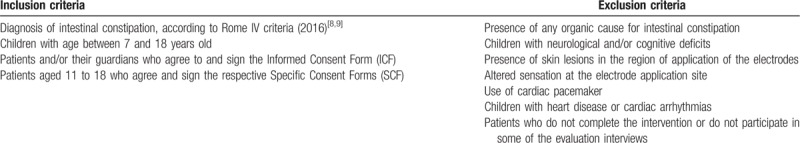
Eligibility criteria.

### 2.5 Intervention

Patients will be submitted to transcutaneous electrical nerve stimulation of the posterior tibial nerve, based on the method described by Queralto et al (2006).^[27]^ This intervention will be supervised by a physiotherapist, who is a member of the research team with experience in this kind of therapy. A silicone auto adhesive electrode (positive electrode) will be applied approximately 3 to 4 cm above the tibial medial malleolus, and a second electrode (negative) will be placed just underneath the medial malleolus of the same leg. The electrodes will be linked to an electrical stimulation device (De Tens/Fes-2 channels, Neurodyn Portable Ibramed). The position of the electrode will be determined by visualization of rhythmic flexion of toes during stimulation. The intensity level will be determined based on the intensity immediately under the maximum sensitive threshold of the patient, usually between 10 and 25 mA. A 200-μs, 30-Hz current will be applied simultaneously in the 2 lower limbs, 30 minutes daily for 4 consecutive weeks, in the patient's home.

First, parents or guardians will be trained to perform PTNS on their children. Training sessions and supervised application will be provided until the parents feel confident enough to perform PTNS without supervision. From that point forward, PTNS will be performed daily in the household, as previously described. During the intervention period, patients should attend a supervised session of PTNS every 15 days, where the technique will be evaluated, and subsequent questions will be clarified. During this period, patients must maintain all therapeutic recommendations (drugs, diets, and behaviors) that had been previously prescribed by their referring physician. Any subsequent changes in treatment during the study period will not be avoided or delayed because of the study. Patients will be instructed to communicate with the research team about any changes in the therapeutic plan during the intervention period.

### 2.6 Adherence to treatment and related adverse events

The research team members will register the treatment of patients in all the biweekly sessions of supervised PTNS and will investigate the possible occurrence of adverse events. At any point during the study, the patients/parents/guardians will be able to contact the research team by telephone to communicate possible adverse events and clarify any doubts. Patients who experience adverse effects or clinical worsening of the evacuatory pattern or who request will be discontinued from the study.

### 2.7 Intervention periods and assessment time points

The intervention period will be 4 consecutive weeks, immediately following the conclusion of the training period. The 3 time points chosen for assessment interviews will be the following: 1 week before the start of the intervention, immediately after the 4 weeks of intervention, and 4 weeks after the end of the intervention period, to identify any remaining effects of PTNS.

### 2.8 Assessment interviews

**Figure 1:**
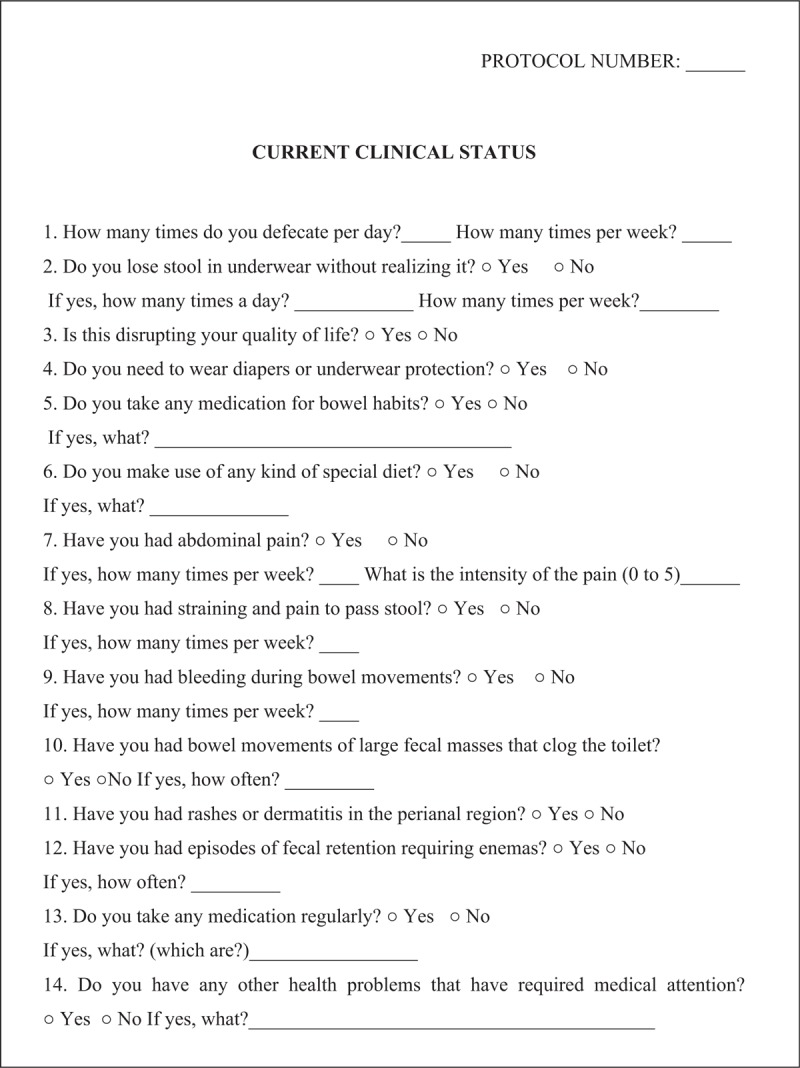
Questionnaire addressing the current clinical status.

**Figure 2:**
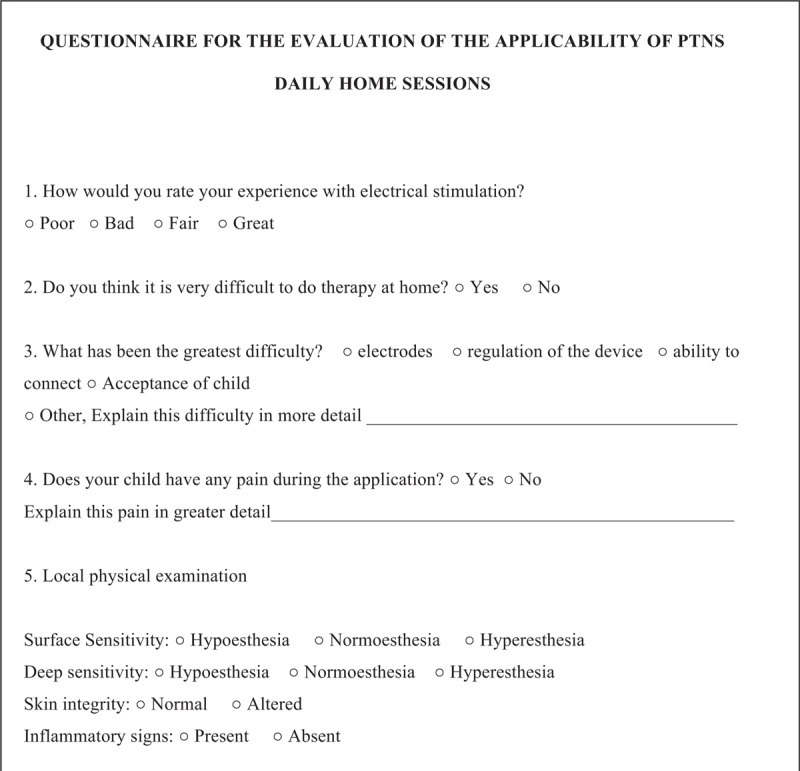
Questionnaire addressing the evaluation of the applicability of the PTNS daily home sessions. PTNS = posterior tibial nerve stimulation.

In all assessment time points, patients and/or their guardians will participate in semi-structured interviews to determine the functional results of the intervention, concerning patient bowel habits and quality of life. These interviews will be conducted by the same member of the research team and will last approximately 40 minutes. The following assessment instruments will be applied: a questionnaire addressing the current clinical status (Fig. 1); the modified Bristol Stool Form Scale for children (mBSFS-C)^[28–30]^ to analyze the stool consistency (see mBSTS-C, Supplemental Digital Content 4); the bowel function score (BF-S)^[31]^ to evaluate the functional status of bowel habits (see BF-S, Supplemental Digital Content 5); the fecal continence index (FCI) questionnaire,^[32,33]^ based on the clinical evaluation of fecal continence (see FCI, Supplemental Digital Content 6); the assessment of quality of life in children and adolescents with fecal incontinence (AQLCAFI)^[32,33]^ (see AQLCAFI, Supplemental Digital Content 7) and the pediatric quality of life inventory version 4.0 (PEDsQL 4.0)^[34,35]^ to assess the quality of life (see PEDsQL 4.0, Supplemental Digital Content 8); and a questionnaire addressing the evaluation of the applicability of the PTNS daily home sessions (Fig. 2).

Additionally, each patient will be monitored throughout a standardized week diary (stool frequency, stool consistency according to the mBSFS-c, frequency of fecal incontinence episodes, painful defecation, abdominal pain, blood in stools, laxative use and their doses, and adverse effects such as pain, nausea, vomiting, diarrhea, and flatulence). This diary must be filled out for 7 days, initiating 7 days before each of the assessment time points. Patients who use laxative medication will have the dosage quantity monitored, and any alterations will be registered as possible outcomes in the conclusion of the study, both in the case of increase or reduction of dosage.

### 2.9 Outcomes

Data analysis will be performed by focusing on the 3 assessment time points: assessment before the initiation of PTNS, immediately after the 4 weeks of intervention, and 4 weeks after the end of the intervention period. This analysis will be performed using the following clinical variables: number of defecations per week, number of episodes of fecal incontinence per week, need for diapers or underwear protection, need for medication for bowel habits, need for a special diet, straining and pain on defecation, presence of fecalomas, presence of abdominal pain, presence of bloody stools, need for enemas, and presence of perianal rashes or dermatitis. Also, we will analyze the results obtained by all the assessment instruments applied (mBSFS-C, BF-S, FCI questionnaire, AQLCAFI, and PEDsQL 4.0), to evaluate the outcomes of a functional status of bowel habits and the global quality of life.

**Figure 3:**
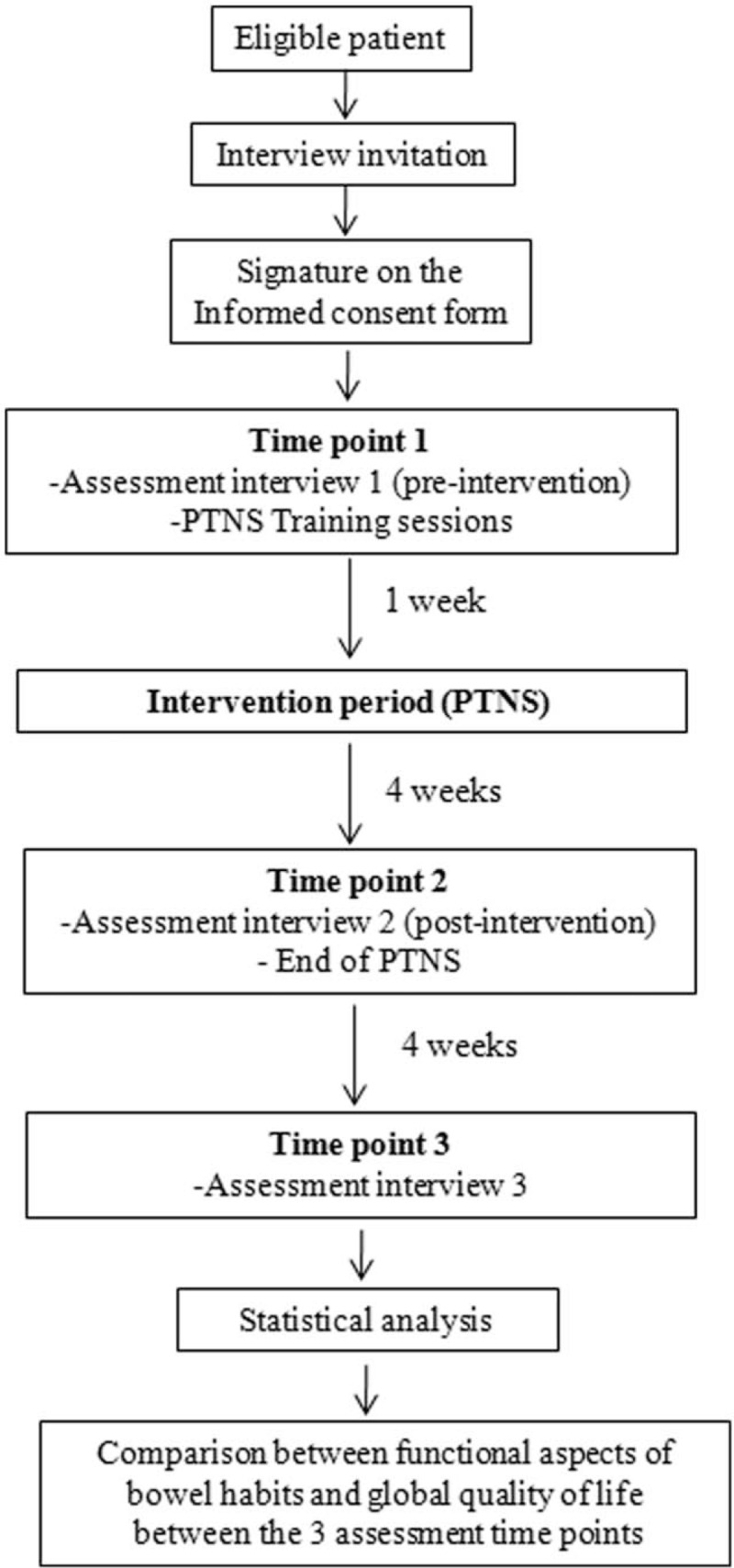
Flowchart of patients in the study.

As a secondary outcome, the applicability of PTNS will also be evaluated by the results of the specific questionnaire (Fig. 3), considering aspects that involve the practical difficulties for the application of this method and possible adverse events. All the situations that may be indirectly involved with the clinical results of PTNS, such as the increase or decrease of the laxative dose or any clinical or dietary changes, will also be analyzed as secondary endpoints.

To summarize the stages of the study, see the flow diagram in Figure 3 and the Supplemental Digital Content 9 (SDC-9) with standard protocol items: recommendations for interventionist tests.

### 2.10 Statistical analysis

Statistical and descriptive analysis will be performed to characterize the patients before the intervention. Then, a comparative statistical analysis will be carried out involving the results obtained for the variables analyzed in the 3 assessment time points. Continuous numerical data will be expressed as the mean ± standard deviation and median (interquartile range). Proportions will be presented as percentages with their respective reliability intervals. The comparison between the assessment time points will be made using different statistical tests, according to variables analyzed and the normality distribution of data obtained by Shapiro–Wilk and Kolmogorov–Smirnov tests. Nominal variables will be analyzed using the McNemar test. Continuous numerical variables of parametric distribution will be analyzed using the *t* test for paired samples and the analysis of variance. Continuous numerical variables of nonparametric distribution and ordinal variables will be analyzed using the Wilcoxon and Friedman tests. The significance level will be established at 5%, and the analysis will be performed using SPSS 22.0 software for Windows.

### 2.11 Protocol amendments

Any amendments to the protocol and information provided to participants will be submitted to the REC for approval before implementation. Substantial amendments may only be implemented after REC approval has been obtained, whereas nonsubstantial amendments can be implemented without written approval from the REC. Data and source documents will be stored such that they can be accessed at a later date for monitoring or inspection by the REC. After the end of the study, the results will be submitted for publication in a peer-reviewed journal, following CONSORT and SQUIRE compliance guidelines. Authorship of any related presentations or reports will be under the name of the collaborative group.

## 3 Discussion

Transcutaneous PTNS has a well-established role in the treatment of children with urinary dysfunction and adults with fecal incontinence.^[20,21,25]^ There is evidence that PTNS can stimulate both the second and third sacral nerve roots, which are the same spinal segments that innervate the bladder, rectum and pelvic floor, thus modulating bowel motility and assisting in the treatment of constipation.^[26,36,37]^ On the other hand, although transcutaneous PTNS can be considered an auspicious, noninvasive and safe method to be used in the pediatric age group, there is still no published study that has investigated its use in children for the treatment of constipation.

The main objective of this study is to assess the applicability and clinical outcomes of transcutaneous PTNS in children with FC. It is intended to demonstrate the efficacy of this promising method to increase the number of complete spontaneous bowel movements, the consistency of stool, to reduce the number of episodes of retentive fecal incontinence, and improve the overall quality of life. This study is currently in the recruitment phase, which began in April 2019.

## Author contributions

**Conceptualization:** Rebeca Mayara Padilha Rego, Nilton Carlos Machado, Mary de Assis Carvalho, Johann Souza Graffunder, Erika Veruska Paiva Ortolan, Pedro Luiz Toledo de Arruda Lourenção.

**Writing – original draft:** Rebeca Mayara Padilha Rego, Nilton Carlos Machado, Mary de Assis Carvalho, Johann Souza Graffunder, Erika Veruska Paiva Ortolan, Pedro Luiz Toledo de Arruda Lourenção.

**Writing – review & editing:** Nilton Carlos Machado, Mary de Assis Carvalho, Erika Veruska Paiva Ortolan, Pedro Luiz Toledo de Arruda Lourenção.

Pedro Luiz Toledo de Arruda Lourenção orcid: 0000-0002-8753-646X.

## Supplementary Material

Supplemental Digital Content
