# Supplementary material for: Transcutaneous posterior tibial nerve stimulation in children and adolescents with functional constipation: A protocol for an interventional study
Source: Medicine (Baltimore). 2019 Nov 11;98(45):e17755. doi: 10.1097/MD.0000000000017755 (PMC6855522; doi:10.1097/MD.0000000000017755)
Supplement: Supplemental Digital Content [file medi-98-e17755-s001.pdf]

UNESP -FACULDADE DE MEDICINA DE BOTUCATU

PARECER CONSUBSTANCIADO DO CEP

DADOS DO PROJETO DE PESQUISA

Título da Pesquisa: Avaliação da aplicabilidade e dos resultados clínicos da Estimulação Elétrica Transcutânea do Nervo Tibial Posterior no tratamento de crianças com constipação intestinal intratável

Pesquisador: REBECA MAYARA PADILHA REGO

Área Temática:

Versão: 1

CAAE: 80013017.0.0000.5411

Instituição Proponente: Faculdade de Medicina de Botucatu/UNESP

Patrocinador Principal: Financiamento Próprio

DADOS DO PARECER

Número do Parecer: 2.420.698

Apresentação do Projeto:

A constipação intestinal crônica (CIC) é considerada afecção extremamente comum na faixa etária pediátrica, sendo responsável por aproximadamente 3% de todas as consultas médicas em pediatria. A grande maioria das crianças com CIC funcional responde ao tratamento padrão. Entretanto, uma parte dos pacientes pode apresentar resposta insatisfatória, com pouca melhora dos sintomas. Essas crianças são classificadas como portadoras de CIC intratável ou refratária. Nestes casos, tratamentos alternativos podem ser empregados. A Estimulação Elétrica Transcutânea do Nervo Tibial Posterior (PTNS) PTNS vem sendo descrita como uma terapia capaz de modular as funções urinárias e evacuatórias, por meio da estimulação dos nervos sacrais. É considerada uma técnica minimamente invasiva, bem tolerada a acessível e que vem sendo cada vez mais utilizada no tratamento de disfunções urinárias na infância. Esta técnica apresenta potencial efeito para a melhora da constipação intestinal. Por tudo isso, nós decidimos avaliar a aplicabilidade e os resultados clínicos da PTNS no tratamento de crianças com constipação intestinal intratável.

O presente projeto propõe a realização de um estudo clínico de intervenção, longitudinal, de coorte, prospectivo, não controlado, cuja coleta de dados ocorrerá no período de 12 meses. A amostra será composta de aproximadamente 30 crianças, com faixa etária entre 7 a 18 anos, que

Endereço: Chácara Butignolli , s/n
Bairro: Rubião Junior CEP: 18.618-970
UF: SP Município: BOTUCATU
Telefone: (14)3880-1609 E-mail: cep@fmb.unesp.br

serão submetidas em ambiente domicilar a estimulação elétrica nervosa transcutânea do nervo tibial posterior em um período de oito semanas, sendo avaliadas antes e após a intervenção. Os resultados esperados são a melhora das variáveis clínicas relacionadas à constipação intestinal crônica, relacionadas ao hábito intestinal, incontinência fecal e qualidade de vida.

Avaliar a aplicabilidade e os resultados clínicos da PTNS no tratamento de crianças com constipação intestinal intratável.

Serão incluídos pacientes com idade entre 7 a 18 anos, de ambos os sexos, com diagnóstico de CIC, estabelecido a partir dos Critérios de Roma IV 8,9, e que sejam classificadas como portadoras de constipação intestinal intratável, de acordo com a definição das Sociedades Européia (ESPGHAN) e Norte Americana (NASPGHAN) de Gastropediatria, representada por pacientes sem resposta ao tratamento padrão, após um período de três meses.

A intervenção programada é a terapia baseada na utilização da corrente de eletroestimulação transcutânea do nervo tibial posterior (PTNS). Esta intervenção será supervisionada por uma fisioterapeuta, membro da equipe de pesquisa, com experiência neste tipo de terapia. Um eletrodo de silicone de 3cm x 5cm (positivo) será aplicado aproximadamente 3-4 cm acima do maléolo medial da tíbia e um segundo eletrodo (negativo) logo abaixo do maléolo medial da mesma perna. Cada sessão de aplicação deverá ter duração de 30 minutos, realizadas nos dois membros inferiores simultaneamente, por meio de um gerador de correntes De Tens/Fes 2 Canais(Neurodyn Portátil Ibramed ®), com frequência de 30 Hz, largura de pulso de 200 μs e intensidade ajustada de acordo com o limiar sensitivo máximo do paciente.Primeiramente, os pais ou responsáveis serão treinados sobre como realizar a PTNS. As sessões de treinamento e aplicação supervisionada serão realizadas até que os pais sintam-se seguros para realizar o procedimento sem supervisão. A partir daí, a PTNS será realizada diariamente, em caráter domicilar, com as mesmas características descritas, por um período de 8 semanas consecutivas. Durante o período de intervenção, os pacientes deverão comparecer a cada 15 dias para uma sessão supervisionada de PTNS, quando será avaliada a técnica empregada nas sessões domiciliares e eventuais dúvidas serão esclarecidas. Neste período, os pacientes deverão, também, manter todas as recomendações terapêuticas (medicamentosas, dietéticas e comportamentais) que já vinham sendo prescritas pela equipe multidisciplinar do ambulatório de distúrbios evacuatórios do HC-FMB-UNESP. Eventuais mudanças necessárias no tratamento, durante o período de estudo, não serão, de maneira alguma,

Endereço: Chácara Butignolli , s/n
Bairro: Rubião Junior CEP: 18.618-970
UF: SP Município: BOTUCATU
Telefone: (14)3880-1609 E-mail: cep@fmb.unesp.br

evitadas ou postergadas por conta do estudo. Os pacientes serão, apenas, orientados a comunicar a equipe de pesquisa sobre qualquer mudança no plano terapêutico proposta pela equipe assistencial, durante o período de intervenção.O período de intervenção será de 8 semanas consecutivas, a partir do momento de término das sessões supervisionadas e início das sessões domiciliares diárias de PTNS. A continuação da terapia com PTNS, após este período de intervenção, ficará na dependência da preferência dos pacientes, pais e/ou responsáveis, após discussão conjunta com a equipe médica responsável. Se houver a opção pela continuidade do tratamento, será realizada uma avaliação dos resultados obtidos após um período de 6 meses. Os momentos de avaliação programados são: - Momento 0 (M0): momento antes do início da intervenção (imediatamente antes da primeira sessão supervisionada de PTNS); - Momento 1 (M1): após as 8 semanas de intervenção;- Momento 2 (M2): 8 semanas após o término da intervenção, nos pacientes que optarem por encerrar o tratamento após o período de intervenção ("washout");- Momento 3 (M3): 6 meses após o início da intervenção, nos pacientes que optarem por manter o tratamento após o período de intervenção. 2.6 Avaliações. Em todos os momentos de avaliação, os pacientes e/ou seus responsáveis irão participar de uma entrevista semi-estruturada quando serão investigados aspectos relacionados ao status funcional do hábito intestinal e qualidade de vida. Essas entrevistas serão conduzidas por uma mesma equipe e terão duração aproximada de 40 minutos. Serão aplicados os seguintes instrumentos de avaliação: - Questionado dirigido para avaliação do estado clínico atual; Escore de Templeton & Ditesheim (1985) para avaliação da incontinência fecal;- Escala de Bristol modificada para crianças (mBSFS-C) para análise da consistência das fezes; Bowel Function Score (BF-S) ; Índice de continência fecal; Questionários PEDs QL 4.0 e QQVCFCA para avaliação de qualidade de vida. Questionário dirigido para avaliação da aplicabilidade das sessões domiciliares diárias de PTNS.

Objetivo da Pesquisa:

Avaliar a aplicabilidade e os resultados clínicos da PTNS no tratamento de crianças com constipação intestinal intratável.

Avaliação dos Riscos e Benefícios:

Riscos relacionados a possibilidade de desconforto local durante a aplicação da eletroestimulação transcutânea e à confidencialidade da identidade dos pacientes, sendo proposta a garantia do sigilo a identidade em eventuais publicações e apresentações científicas. Benefícios: A possibilidade de melhorar os sintomas de constipação intestinal, como por exemplo, o aumento do número de evacuações e a diminuição da dor e do esforço para evacuar.

Endereço: Chácara Butignolli , s/n
Bairro: Rubião Junior CEP: 18.618-970
UF: SP Município: BOTUCATU
Telefone: (14)3880-1609 E-mail: cep@fmb.unesp.br

Comentários e Considerações sobre a Pesquisa:

O projeto apresenta boa justificativa teórica para sua realização. Os objetivos são claros e a metodologia está bem detalhada.

É um estudo de intervenção, porém a terapêutica proposta já é utilizada como opção para casos selecionados, carecendo, segundo os autores, de mais estudos que mostrem sua aplicabilidade e resolutividade.

Considerações sobre os Termos de apresentação obrigatória:

Todos os termos e declarações foram anexados, incluindo Termos de Assentimento e TCLEs direcionados aos pais e aos maires de 12 anos.

Os termos são claros, de fácil compreensão e elucidativos.

Recomendações:

Recomendo que os autores esclareçam como será a logística de "empréstimo" dos equipamentos para eletroestimulação domicilar, principalmente nos casos onde o tratamento for mantido após a descontinuação da pesquisa.

Conclusões ou Pendências e Lista de Inadequações:

Diante do exposto, sou de parecer favorável à aprovação do projeto, sem necessidade de envio à CONEP. Em relação à recomendação por mim feita, não acredito que isso tenha implicação ética limitante para a realização da pesquisa.

Considerações Finais a critério do CEP:

Conforme deliberação do Colegiado em reunião ordinária do Comitê de Ética em Pesquisa da FMB/UNESP, realizada em 04 dezembro de 2017, o projeto encontra-se APROVADO, sem necessidade de envio à CONEP.

O Comitê de Ética em Pesquisa, no entanto, informa que ao final da execução da pesquisa, seja enviado o "Relatório Final de Atividades", na forma de "Notificação", via Plataforma Brasil.

Atenciosamente,

Comitê de Ética em Pesquisa da Faculdade de Medicina de Botucatu - UNESP

Este parecer foi elaborado baseado nos documentos abaixo relacionados:

Tipo Documento	Arquivo	Postagem	Autor	Situação
Informações Básicas do Projeto	PB_INFORMAÇÕES_BÁSICAS_DO_PROJETO_992129.pdf	10/11/2017 12:52:12		Aceito
Outros	anuencia_instituic_rebeca.pdf	10/11/2017 12:51:57	Pedro Luiz Toledo de Arruda Lourenção	Aceito
TCLE / Termos de Assentimento / Justificativa de Ausência	T_assentimento.docx	10/11/2017 12:51:11	Pedro Luiz Toledo de Arruda Lourenção	Aceito
TCLE / Termos de Assentimento / Justificativa de Ausência	TCLE_pais_maiores_12.docx	10/11/2017 12:51:03	Pedro Luiz Toledo de Arruda Lourenção	Aceito
TCLE / Termos de Assentimento / Justificativa de Ausência	TCLE_pais_menores_11.docx	10/11/2017 12:50:51	Pedro Luiz Toledo de Arruda Lourenção	Aceito
Projeto Detalhado / Brochura Investigador	projeto_rebeca.pdf	10/11/2017 12:44:30	Pedro Luiz Toledo de Arruda Lourenção	Aceito
Folha de Rosto	folha_rosto_rebeca.Pdf	10/11/2017 12:43:12	Pedro Luiz Toledo de Arruda Lourenção	Aceito

Situação do Parecer:
Aprovado

Necessita Apreciação da CONEP:
Não

BOTUCATU, 07 de Dezembro de 2017

Assinado por:
SILVANA ANDREA MOLINA LIMA
(Coordenador)

Endereço: Chácara Butignoli , s/n
Bairro: Rubião Junior **CEP:** 18.618-970
UF: SP **Município:** BOTUCATU
Telefone: (14)3880-1609 **E-mail:** cep@fmb.unesp.br

TERMO DE CONSENTIMENTO LIVRE E ESCLARECIDO

CONVIDO, o Senhor (a),______________________________________ responsável pelo(a) menor ____ para participar do Projeto de Pesquisa intitulado "Avaliação da aplicabilidade e dos resultados clínicos da Estimulação Elétrica Transcutânea do Nervo Tibial Posterior no tratamento de crianças com constipação intestinal intratável",que será desenvolvido por mim Rebeca Mayara Padilha Rego, Fisioterapeuta, com orientação do profissional Medico e Professor (a) Dr. Pedro Luiz Toledo de Arruda Lourenção Lourenção, da Faculdade de Medicina de Botucatu –UNESP.

Nós estamos investigando a utilização de uma nova terapia para crianças com constipação intestinal grave, como é o caso do seu (ua) filho(a). Esta nova terapia acontece pela estimulação nervosa de um nervo da perna, que consegue estimular o intestino a funcionar melhor. Alguns estudos já demonstraram que esse tratamento pode ser eficaz, mas nós decidimos estudá-lo para conhecer melhor os seus possíveis resultados.

Se o(a) senhor(a) desejar participar do estudo, será preciso que o(a) senhor(a) e seu (ua) filho(a) compareçam para as avaliações agendadas, a cada 15 dias e que você aplique, em casa, a eletroestimulação nas duas pernas do(a) seu(ua) filho(a), por 30 minutos, todos os dias, em um período de 8 semanas. A criança, durante a estimulação da perna, não deve sentir dor, mas pode ser que apresente uma sensação parecida como se tivessem varias formiguinhas passando de um lado para o outro na perna. Antes de realizar essa estimulação em casa, você terá um treinamento e só fara a eletro estimulação em casa quando se sentir seguro o suficiente.

Será preciso, também, que uma semana antes de toda avaliação marcada, o(a) senhor(a) preencha um diário que vamos te fornecer, relacionado ao hábito intestinal da criança. São informações sobre frequência em que foi no banheiro, forma e consistência do cocô e episódios de perdas fecais. Eu entrarei em contato por telefone para recordá-lo (a) sobre o preenchimento e o (a) senhor (a) poderá entrar em contato com a equipe de pesquisa caso tenha alguma duvida ou para relatar algum evento adverso. Em 3 das consultas que vocês vierem, durante o período do estudo, também serão necessários que o(a) senhor(a) e seu (ua) filho(a) respondam a algumas perguntas e preencham alguns questionários, relacionados a aspectos intestinais e sobre a qualidade de vida. Nestas 3 vezes, as consultas serão um pouco mais demoradas, e devem durar aproximadamente 30 minutos.

Solicito também seu consentimento para levantar o prontuário médico do seu filho (a) para coletar informações lá contidas como os sintomas mais comuns e os tratamentos que vem sendo realizados, referentes a consultas feitas anteriormente.

O benefício que seu filho (a) terá em participar será a possibilidade de melhorar os sintomas de constipação intestinal, como por exemplo, o aumento da frequência evacuatória e a redução de dor ou dificuldade ao evacuar e melhora da qualidade de vida. Como se trata de uma pesquisa, não podemos garantir que este tratamento traga, com total certeza, esses benefícios. Ao termino do estudo, se o(a) senhora (a) acharem que o tratamento está fazendo bem para a criança e quiserem continuar o tratamento, o tratamento será mantido até completar 6 meses de duração, e após este período, o quadro clínico será novamente reavaliado.

Fique ciente que a participação do seu filho neste estudo é voluntária e que mesmo após ter dado seu consentimento para participar da pesquisa, você poderá retirar a qualquer momento, sem qualquer prejuízo na continuidade do tratamento do seu filho(a).

Este Termo de Consentimento Livre e Esclarecido será elaborado em 2 vias de igual teor, o qual 01 via será entregue ao Senhor (a) devidamente rubricada, e a outra via será arquivada e mantida pelos pesquisadores por um período de 5 anos após o término da pesquisa.

Qualquer dúvida adicional você poderá entrar em contrato com o Comitê de Ética em Pesquisa através dos telefones (14) 3880-1608 ou 3880-1609 que funciona de 2ª a 6ª feira das 8.00 às 11.30 e das 14.00 às 17horas, na Chácara Butignolli s/nº em Rubião Júnior – Botucatu - São Paulo. Os dados de localização dos pesquisadores estão abaixo descrito:

Após terem sido sanadas todas minhas dúvidas a respeito deste estudo, CONCORDO que meu (minha) filho (a) participe de forma voluntária, estando ciente que todos os seus dados estarão resguardos através do sigilo que os pesquisadores se comprometeram. Estou ciente que os resultados desse estudo poderão ser publicados em revistas científicas, sem, no entanto, que a identidade minha e do(a) meu(inha) filho(a) seja revelada.

Botucatu,_____/___/______

_______________________ ___________________________

Pesquisador Responsável pelo Participante da Pesquisa

Rebeca Mayara Padilha Rego

Endereço: Departamento de Cirurgia e Ortopedia – Anexo Verde – 3º andar. Faculdade de Medicina de Botucatu – UNESP. Av. Prof. Mário Rubens Guimarães Montenegro, s/n. Bairro: UNESP - Campus de Botucatu. CEP: 18618-687 - Botucatu, SP

Telefone: (14) 3880-1703

Email: rebecamayara.fisio@gmail.com

Orientador: Prof. Dr. Pedro Luiz Toledo de Arruda Lourenção

Endereço: Departamento de Cirurgia e Ortopedia – Anexo Verde – 3º andar. Faculdade de Medicina de Botucatu – UNESP. Av. Prof. Mário Rubens Guimarães Montenegro, s/n. Bairro: UNESP - Campus de Botucatu. CEP: 18618-687 - Botucatu, SP

Telefone: (14) 3880-1703

Email: lourencao@fmb.unesp.br

TERMO DE ASSENTIMENTO LIVRE E ESCLARECIDO (TALE)

RESOLUÇÃO 466/2012

Para menores entre 12 a 17 anos/11meses e 29 dias.

CONVIDO, você,____ para participar do Projeto de Pesquisa intitulado "Avaliação da aplicabilidade e dos resultados clínicos da Estimulação Elétrica Transcutânea do Nervo Tibial Posterior no tratamento de crianças com constipação intestinal intratável", que será desenvolvido por mim Rebeca Mayara Padilha Rego, Fisioterapeuta, com orientação do profissional Medico e Professor (a) Dr. Pedro Lourenção da Faculdade de Medicina de Botucatu –UNESP.

Caso concorde em participar da pesquisa você deverá assinar este Termo de Assentimento e seu Representante Legal assinará o Termo de Consentimento Livre e Esclarecido para participantes de pesquisa entre 11 a 17 anos/11/meses e 29 dias.

Nós estamos investigando a utilização de uma nova terapia para pessoas com constipação intestinal grave, como você. Esta nova terapia acontece pela estimulação nervosa de um nervo da perna, que consegue estimular o intestino a funcionar melhor. Alguns estudos já demonstraram que esse tratamento pode ser eficaz, mas nós decidimos estudá-lo para conhecer melhor os seus possíveis resultados.

Se você desejar participar do estudo, será preciso que você compareça, acompanhado por seu(ua) responsável, para as avaliações agendadas, a cada 15 dias e que você aplique, em casa, a eletroestimulação nas suas duas pernas por 30 minutos, todos os dias, em um período de 8 semanas. Durante a estimulação da perna você não deverá sentir dor, mas pode ser que você sinta uma sensação parecida como se tivessem varias formiguinhas passando de um lado para o outro na sua perna. Antes de realizar essa estimulação em casa, você e seu (ua) responsável terão um treinamento e só irão iniciar a eletro estimulação em casa quando se sentirem seguros para isso.

Será preciso, também, que uma semana antes de toda avaliação marcada, vocês preencham um diário que vamos te fornecer, relacionado ao seu hábito intestinal. São informações sobre o número de vezes que você está fazendo cocô, a forma e a consistência do cocô e episódios de perdas fecais. Eu entrarei em contato por telefone para recordá-los (a) sobre o preenchimento

e o você e seu(ua) responsável poderão entrar em contato com a equipe de pesquisa caso tenha alguma duvida ou para relatar algum evento adverso. Em 3 das consultas que vocês vierem, durante o período do estudo, também serão necessários que vocês respondam a algumas perguntas e preencham alguns questionários, relacionados a aspectos intestinais e sobre a qualidade de vida. Nestas 3 vezes, as consultas serão um pouco mais demoradas, e devem durar aproximadamente 30 minutos.

Solicito também seu consentimento para levantar dados do seu prontuário médico para coletar informações lá contidas, como os sintomas mais comuns e os tratamentos que vem sendo realizados, referentes a consultas feitas anteriormente.

O benefício que você terá em participar será a possibilidade de melhorar os seus sintomas de constipação intestinal, como por exemplo, o aumento do número de vezes que você faz cocô e a diminuição da dor e do esforço para evacuar. Como se trata de uma pesquisa, não podemos garantir que este tratamento traga, com total certeza, esses benefícios. Ao termino do estudo, se você e sdeu(ua) responsável acharem que o tratamento está fazendo bem para você e quiserem continuar o tratamento, o tratamento será mantido até completar 6 meses de duração, e após este período, o quadro clínico será novamente reavaliado.

Fique ciente, que a participação neste estudo é voluntária e que mesmo após ter dado consentimento para participar da pesquisa, você poderá retirar a qualquer momento, sem qualquer prejuízo na continuidade do tratamento, ou qualquer outra atividade em que você esteja participando.

Este Termo de Assentimento Livre e Esclarecido será elaborado em 2 vias de igual teor, o qual 01 via será entregue a você devidamente rubricada, e a outra via será arquivada e mantida pelos pesquisadores por um período de 5 anos após o término da pesquisa.

Qualquer dúvida adicional você poderá entrar em contrato com o Comitê de Ética em Pesquisa através dos telefones (14) 3880-1608 ou 3880-1609 que funciona de 2ª a 6ª feira das 8.00 às 11.30 e das 14.00 às 17horas, na Chácara Butignolli s/nº em Rubião Júnior – Botucatu - São Paulo. Os dados de localização dos pesquisadores estão abaixo descrito:

Após terem sido sanadas todas minhas dúvidas a respeito deste estudo, CONCORDO em participar de forma voluntária, estando ciente que todos os meus dados estarão resguardos através do sigilo que os pesquisadores se comprometeram. Estou ciente que os resultados desse estudo poderão ser publicados e revistas científicas, sem que a minha identidade seja revelada.

Botucatu,_____/___/______

__________________________ ____________________________

Pesquisador **Participante da Pesquisa**

Rebeca Mayara Padilha Rego

Endereço: Departamento de Cirurgia e Ortopedia – Anexo Verde – 3º andar. Faculdade de Medicina de Botucatu – UNESP. Av. Prof. Mário Rubens Guimarães Montenegro, s/n. Bairro: UNESP - Campus de Botucatu. CEP: 18618-687 - Botucatu, SP

Telefone: (14) 3880-1703

Email: rebecamayara.fisio@gmail.com

Orientador: Prof. Dr. Pedro Luiz Toledo de Arruda Lourenção

Endereço: Departamento de Cirurgia e Ortopedia – Anexo Verde – 3º andar. Faculdade de Medicina de Botucatu – UNESP. Av. Prof. Mário Rubens Guimarães Montenegro, s/n. Bairro: UNESP - Campus de Botucatu. CEP: 18618-687 - Botucatu, SP

Telefone: (14) 3880-1703

Email: lourencao@fmb.unesp.br

modified Bristol Stool Form Scale for Children – m-BSFS-C

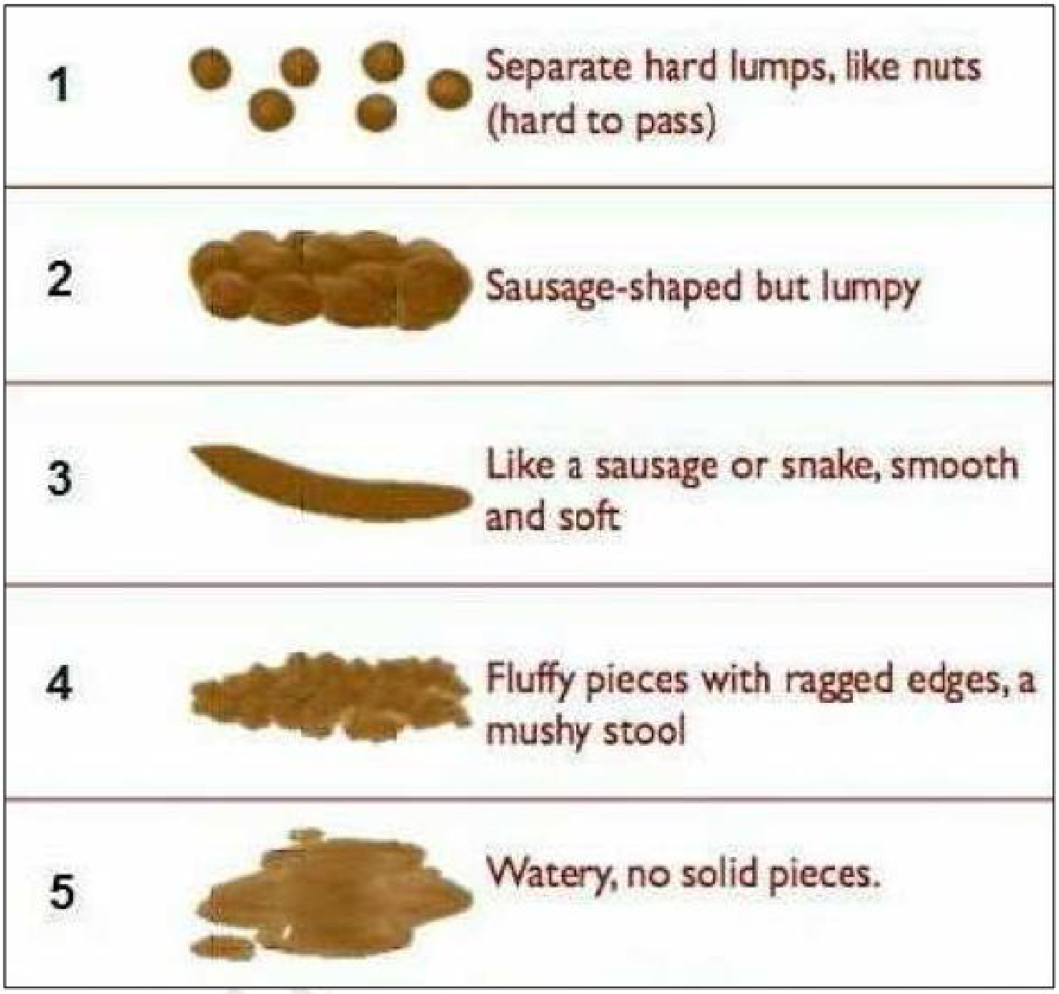

-Chumpitazi BP, Lane MM, Czyzewski DI, Weidler EM, Swank PR, Shulman RJ. Creation and initial evaluation of a stool form scale for children. J Pediatr. 2010;157: 594-597.

-Lane MM, Czyzewski DI, Chumpitazi BP, Shulman RJ. Reliability and validity of a modified Bristol Stool Form Scale for children. J Pediatr. 2011;159:437-441.

-Jozala DR, Oliveira ISF, Ortolan EVP, et al. Brazilian Portuguese translation, cross-cultural adaptation and reproducibility assessment of the modified Bristol Stool Form Scale for children. J Pediatr (Rio J). 2018 Mar 15. pii: S0021-7557(17)31151-8.

Bowel Function Score

Factor	Score Given
Ability to hold back defecation	
Always	3
Problems <1/week	2
Weekly problems	1
No voluntary control	0
Feels the urge to defecate	
Always	3
Most of the time	2
Uncertain	1
Absent	0
Frequency of defecation	
Every other day—twice a day	2
More often	1
Less often	1
Soiling	
Never	3
Staining <1/week, no change of underwear required	2
Frequent staining/soiling, change of underwear required	1
Daily soiling, requires protective aids	0
Accidents	
Never	3
Less than 1/week	2
Weekly accidents, often requires protective aids	1
Daily, protective aids required day and night	0
Constipation	
No constipation	3
Manageable with diet	2
Manageable with laxatives	1
Manageable with enemas	0
Social problems	
No social problems	3
Sometimes (foul odors)	2
Problems causing restrictions of social life	1
Major social/psychological problems	0

Adapted from

Jarvi K, Laitakari EM, Koivusalo A, Rintala RJ, Pakarinen MP. Bowel function and gastrointestinal quality of life among adults operated for Hirschsprung disease during childhood: a population-based study. Ann Surg. 2010; 252(6):977-981.

Fecal Continence Index (FCI) questionnaire.

A. Identification

Name:

Address: Neighborhood: City:

State: Postal Code:

Telephone numbers:

B. Introduction

1. Who is answering?
   a) Self;
   b) Assisted by another person;
   c) Answered by another person.
2. Current age: ___ Gender: __________
3. What is your main activity?
   a) Student.
      a. In what grade are you? ____________
   b) Worker.
4. In the past, you have had a surgery to correct an intestinal malformation. In addition to this problem, have you had any other health problem that required medical treatment or surgery? If yes, which one(s)?
5. Do these other problems still cause you difficulties? If yes, which one(s)?

C. Control of bowel movements (stool)

Some patients with intestinal malformations may have difficulty in controlling bowel movements (stool) even after undergoing surgical correction. Our objective is to understand, through this questionnaire, if your intestine troubles you and how often. If you experience the problem mentioned in this question, but not because of difficulty with bowel movements, please leave the answer blank.

1. How often do you evacuate in a toilet in a full day (24 hours)? ______
2. At night, after going to bed, how often do you need to get up due to bowel movements? _____
3. In a full day (24 hours), how often do you need to change clothes, boxers or panties because they are soiled with feces? ______ If you do not soil your clothes every day, how many times per week or per month does this happen? ______
4. If you need to leave or have an appointment, which of the following alternative(s) do you usually follow to stay clean?
   a) Use a liner, diaper, or change into extra clothes.
   b) Use an enema and/or avoid eating before leaving.
   c) Go to the toilet before leaving.
   d) I do not make any preparation before leaving.
5. If you have time and are willing to do all of the preparations before leaving, what is the result?
   a) Stay clean for over 4 hours.
   b) Stay clean up to 4 hours.
   c) Preparation does not help because I soil myself unintentionally.
6. Do you usually have diarrhea? How often?
   a) Every week.
   b) 1 to 2 times per month.
   c) Never.
7. How often are you constipated, going days without a bowel movement, with stool forming hard balls in the belly that require removal through maneuvers in the anus?
   d) Every week.
   e) 1 time per month.
   f) Never.
8. Check the care you take regularly in your routine to improve bowel function:
   a) Do not eat certain foods because they cause me harm.
   b) Eat at regular times in order to go to the toilet after meals.
   c) Give myself an enema.
   d) Take medications. Which one(s)? ______

-Tannuri AC, Ferreira MA, Mathias AL, Tannuri U. Long-term results of the Duhamel technique are superior to those of the transanal pullthrough: A study of fecal continence and quality of life. J Pediatr Surg. 2017 Mar;52(3):449-453.
- Mathias AL, Tannuri AC, Ferreira MA, Santos MM, Tannuri U. Validation of questionnaires to assess quality of life related to fecal incontinence in children with anorectal malformations and Hirschsprung's disease. Rev Paul Pediatr. 2016 Jan-Mar;34(1):99-105.

Assessment of Quality of Life in Children and Adolescents with Fecal Incontinence (AQLCAFI).

A. Individual and social questions

Below, we will ask other questions to determine if your bowel function bothers you and how often it bothers you. If this complaint exists but it is not because of poor bowel function, please leave the question blank.

	Almost always	Sometimes	Rarely	Never
1 When I am away from home, I try to stay near the bathroom				
2 I avoid visiting my friends				
3 I am not often invited to parties or to go on trips				
4 I avoid spending the night away from home				
5 I fear that people may smell feces or flatus				
6 I would rather stay home than go out				
7 I avoid eating out				
8 I am unable to participate in activities with my friends				
9 I avoid talking about the problem with others				
10 I need to plan my activities according to my bowel function				
11 It impairs my school performance				
12 It affects my professional work				
13 I release stool without noticing				
14 I prefer for people not to be aware of my problem				
15 It hinders the practice of sports				
16 I avoid traveling				
17 I have a difficult time making friends				
18 I worry about accidents with feces				
19 Leaving home worries me				
20 I stop doing the things I like				
21 I feel that I cannot control my bowel movements				
	Poor	Fair	Good	Excellent
22 In general, you think your health is:				
23 How would you rate your bowel function?				
24 In your case, if it was indicated, would you accept other forms of treatment or surgeries to improve your bowel function?				

-Tannuri AC, Ferreira MA, Mathias AL, Tannuri U. Long-term results of the Duhamel technique are superior to those of the transanal pullthrough: A study of fecal continence and quality of life. J Pediatr Surg. 2017 Mar;52(3):449-453.
- Mathias AL, Tannuri AC, Ferreira MA, Santos MM, Tannuri U. Validation of questionnaires to assess quality of life related to fecal incontinence in children with anorectal malformations and Hirschsprung's disease. Rev Paul Pediatr. 2016 Jan-Mar;34(1):99-105.

User agreement

Special Terms

Mapi Research Trust, a non-for-profit organisation subject to the terms of the French law of 1st July 1901, registered in Carpentras under number 453 979 346, whose business address is 27 rue de la Villette, 69003 Lyon, France, hereafter referred to as "MRT" and the User, as defined herein, (each referred to singularly as a "Party" and/or collectively as the "Parties"), do hereby agree to the following User Agreement Special and General Terms:

Mapi Research Trust
PROVIDE™
27 rue de la Villette
69003 Lyon
France
Phone: +33 (0)4 72 13 66 66

Recitals

The User acknowledges that it is subject to these Special Terms and to the General Terms of the Agreement, which are included in Appendix 1 to these Special Terms and fully incorporated herein by reference. Under the Agreement, the Questionnaire referenced herein is licensed, not sold, to the User by MRT for use only in accordance with the terms and conditions defined herein. MRT reserves all rights not expressly granted to the User.

The Parties, in these Special Terms, intend to detail the special conditions of their partnership.

The Parties intend that all capitalized terms in the Special Terms have the same definitions as those given in article 1 of the General Terms included in Appendix 1.

In this respect, the Parties have agreed as follows:

Article 1. Conditions Specific to the User

Section 1.01 Identification of the User

User Name	Pedro Luiz Toledo de Arruda Lourenção
Legal Form	University/Hospital
Address	Av. Prof. Mário Rubens Guimarães Montenegro, s/n<br>Departamento de Cirurgia e Ortopedia<br>18618687 Botucatu
Country	Brazil
Email address	plourencao@gmail.com
Telephone number	+551438801703

Section 1.02 Identification of the Questionnaire

© Mapi Research Trust. The unauthorized modification and use of any portion of this document is prohibited.

Title	Pediatric Quality of Life Inventory™ (PedsQL™)
Author(s)	Varni JW
Owner	Varni James W, PhD
Copyright	Copyright © 1998 JW Varni, Ph.D. All rights reserved
Original bibliographic references	See Appendix 2

Article 2. Rights to Use

Section 2.01 Context of the Use of the Questionnaire

The User undertakes to only use the Questionnaire in the context of the Study as defined hereafter.

Context of Use	Clinical project or study
Title	Evaluation of the initial impact of the transcutaneous Posterior Tibial Nerve Stimulation in children with intestinal constipation: protocol for an interventional study.
Disease or condition	intestinal constipation
Type of research	Clinical trial
Study/Protocol reference	The Brazilian Registry of Clinical Trials (Rebec) identifier for this study is RBR-344jq8, obtained on march 13, 2018 (Número do UTN: U1111-1207-5487), available at http://www.ensaiosclinicos.gov.br.
Number of patients expected	28
Number of submissions to the questionnaire for each patient	1
Term of clinical follow-up for each patient	Yes
Start	04/2019
End	04/2020
Mode of administration	Paper administration

Section 2.02 Conditions for Use

The User undertakes to use the Questionnaire in accordance with the conditions for use defined hereafter.

(a) Rights transferred
Acting in the Owner's name, MRT transfers the following limited, non-exclusive rights, to the User (the "Limited Rights")

(i) to use the Questionnaire, only as part of the Study; this right is made up exclusively of the right to communicate it to the Beneficiaries only, free of charge, by any means of communication and by any means of remote distribution known or unknown to date, subject to respecting the conditions for use described hereafter; and

(ii) to reproduce the Questionnaire, only as part of the Study; this right is made up exclusively of the right to physically establish the Questionnaire or to have it physically established, on any paper, electronic, analog or digital medium, and in particular documents, articles, studies, observations, publications, websites whether or not protected by restricted access, CD, DVD, CD-ROM, hard disk, USB flash drive, for the Beneficiaries only and subject to respecting the conditions for use described hereafter; and

© Mapi Research Trust. The unauthorized modification and use of any portion of this document is prohibited.

(iii) Should the Questionnaire not already have been translated into the language requested, the User is entitled to translate the Questionnaire or have it translated in this language, subject to informing MRT of the same beforehand by the signature of a Translation Agreement indicating the terms of it and to providing a copy of the translation thus obtained as soon as possible to MRT.

The User acknowledges and accepts that it is not entitled to amend, modify, condense, adapt, reorganise the Questionnaire on any medium whatsoever, in any way whatsoever, even minor, without MRT's prior specific written consent.

(b) Specific conditions for the Owner

The Owner has intended to transfer a part of the copyright on the Questionnaire and/or the Documentation to MRT in order to enable MRT to make it available to the User for the purpose of the Study, subject to the User respecting the following conditions:

User shall not modify, abridge, condense, translate, adapt, recast or transform the Questionnaire in any manner or form, including but not limited to any minor or significant change in wordings or organisation in the Questionnaire, without the prior written agreement of the Owner. If permission is granted, any improvements, modifications, or enhancements to the Questionnaire which may be conceived or developed, including translations and modules, shall become the property of the Owner.

The User therefore undertakes to respect these special terms.

(c) Specific conditions for the Questionnaire

- Use in Individual clinical practice or Research study / project

The User undertakes never to duplicate, transfer or publish the Questionnaire without indicating the Copyright Notice.

In the case of use of an electronic version of the Questionnaire in academic studies, the User undertakes to respect the following special obligations:

- In case of use of an IT Company (e-vendor), User shall check with Mapi Research Trust that IT Company has signed the necessary License Agreement with Mapi Research Trust before developing the electronic version of the Questionnaire
- Not modify the questionnaire (items and response scales, including the response scale numbers from 0-4)
- Cite the reference publications
- Insert the Owner's copyright notice on all pages/screens on which the Questionnaire will be presented and insert the Trademark information: PedsQL™, Copyright © 1998 JW Varni, Ph.D. All rights reserved.
- Mention the following information: "PedsQL™ contact information and permission to use: Mapi Research Trust, Lyon, France – Internet: https://eprovide.mapi-trust.org and www.pedsql.org/index.html "
- Submit the screenshots of all the Pages where the Questionnaire appears to Dr James W. Varni before release for approval and to check that the above-mentioned requirements have been respected.

In the case of use of an electronic version of the Questionnaire in commercial studies / projects, the User undertakes to respect the following special obligations:

User shall:

- In case of use of an IT Company (e-vendor), User shall check with Mapi Research Trust that IT Company has signed the necessary License Agreement with Mapi Research Trust before developing the electronic version of the Questionnaire
- Not modify the questionnaire (items and response scales, including the response scale numbers from 0-4)
- Cite the reference publications
- Insert the Owner's copyright notice on all pages/screens on which the Questionnaire will be presented and insert the Trademark information: PedsQL™, Copyright © 1998 JW Varni, Ph.D. All rights reserved.
- Mention the following information: "PedsQL™ contact information and permission to use: Mapi Research Trust, Lyon, France – Internet: https://eprovide.mapi-trust.org and www.pedsql.org/index.html"

© Mapi Research Trust. The unauthorized modification and use of any portion of this document is prohibited.

- For the first migration of the Questionnaire (generally the original version) into a specific electronic device

  - Review of screenshots:

After implementation of the Questionnaire into the device, the user/IT Company will generate screen captures (screenshots) of the original questionnaire as displayed in the device. These will be reviewed by Mapi to check that they are consistent with the original paper version in terms of presentation, content and completion except for specific instructions related to the electronic administration. Corrections that may be needed will be reported to the user/IT Company. In this case, screenshots after correction will be generated for another round of review by Mapi until all screenshots are approved.

Dr James W. Varni will review all approved screenshots for a final validation.

  - Usability testing:

Usability testing is a methodology which aims to examine whether respondents are able to use a device and associated software as intended. Major issues of concern in usability testing typically include device complexity, navigation and response selection for example.

The objective of this investigation is to ensure that the electronic version of the questionnaire as included in the device meets usability criteria, focusing on functional aspects and respondents' understanding of instructions. Usability testing consists in interviews with patients where patients will complete the electronic version of the Questionnaire on the device and comment on their understanding of the instructions, ease of use and handiness of the device. A Usability testing report presenting results will be produced. If any changes are recommended, these will be implemented by the user/IT Company. If issues raised by respondents are rated as major, the user/IT Company may need to perform additional developments and another round of interviews may be needed.

Dr James W. Varni will review the changes suggested, if any, following the interviews.

The review of screenshots is mandatory. The usability testing is highly recommended by Mapi, however should the User and/or IT Company decide not to perform this step, Mapi Research Trust shall not be held responsible for any consequence and expense associated with this decision which shall remain the User and/or IT Company's sole liability.

The review of screenshots and usability testing, when and if performed, shall be performed exclusively by Mapi and shall be sponsored by the User.

The performance of the review of screenshots and usability testing will result in a certification of the electronic device original version of the Questionnaires by Mapi for future licenses.

- For the migration of other language versions of the Questionnaire on an existing certified specific electronic device

  - Update version

After the electronic device original version of the Questionnaire is fully ready, the Questionnaire's language versions developed for paper administration will be updated to reflect the changes in wording of instructions implemented in the electronic device original version of the questionnaire.

Native speakers of the languages will reflect the changes made to the electronic device original version of the Questionnaire and will provide English equivalents of all changes made for Mapi's quality control.

  - Review of screenshots:

© Mapi Research Trust. The unauthorized modification and use of any portion of this document is prohibited.

After implementation of the Questionnaire into the device, the user/IT Company will generate screen captures (screenshots) of the original questionnaire as displayed in the device. These will be reviewed by Mapi to check that they are consistent with the original paper version in terms of presentation, content and completion except for specific instructions related to the electronic administration. Corrections that may be needed will be reported to the user/IT Company. In this case, screenshots after correction will be generated for another round of review by Mapi until all screenshots are approved.

The update of version and review of screenshots are mandatory. These steps shall be performed exclusively by Mapi and shall be sponsored by the User.

The performance of the update of version and review of screenshots will result in a certification of the electronic device language version of the Questionnaires by Mapi for future licenses.

- Use in a publication:

In the case of a publication, article, study or observation on paper or electronic format of the Questionnaire, the User undertakes to respect the following special obligations:

- not to include any full copy of the Questionnaire, but a version with the indication "sample copy, do not use without permission"
- to indicate the name and copyright notice of the Owner (PedsQL™, Copyright © 1998 JW Varni, Ph.D. All rights reserved)
- to include the reference publications of the Questionnaire
- to indicate the details of MRT for any information on the Questionnaire as follows: "PedsQL™ contact information and permission to use: Mapi Research Trust, Lyon, France. – Internet: https://eprovide.mapi-trust.org and www.pedsql.org "
- to provide MRT, as soon as possible, with a copy of any publication regarding the Questionnaire, for information purposes
- to submit the screenshots of all the Pages where the Questionnaire appears to MRT before release to check that the above-mentioned requirements have been respected.

- Use for dissemination or marketing:

In the case of use in a dissemination/marketing context:

- On a website with unrestricted access:

The publication of a copy of the PedsQL™ on a website with unrestricted access is not permitted.

- On a website with restricted access:

In the case of publication on a website with restricted access, the User may include a version of the Questionnaire that may be amended, subject to this version being protected by a sufficiently secure access to only allow the Beneficiaries to access it.

Article 3. Term

MRT transfers the Limited Rights to use the Questionnaire as from the date of delivery of the Questionnaire to the User and for the whole period of the Study.

Article 4. Beneficiaries

© Mapi Research Trust. The unauthorized modification and use of any portion of this document is prohibited.

The Parties agree that the User may communicate the Questionnaire in accordance with the conditions defined above to the Beneficiaries involved in the Study only, in relation to the Study defined in section 2.01.

Article 5. Territories and Languages

MRT transfers the Limited Rights to use the Questionnaire on the following territories and in the languages indicated in the table below:

Questionnaire	Language
PedsQL™ Generic Core Scales	Portuguese for Brazil

Article 6. Price and Payment Terms

The User undertakes in relation to MRT to pay the price owed in return for the availability of the Questionnaire, according to the prices set out below, depending on the languages requested and the costs of using the Questionnaire, in accordance with the terms and conditions described in section 6.02 of the General Terms included in Appendix 1.

Access to the Questionnaire in non-funded academic research and individual clinical practice is free of charge.

Agreed and acknowledged by

Pedro Luiz Toledo de Arruda Lourenção

21-Jan-2019

Appendix 1 to the Special Terms: User Agreement General Terms

User has read and accepted the Mapi's General Terms of the Agreement, which are available on MRT's website: https://eprovide.mapi-trust.org/user-agreement-general-terms

Appendix 2 to the Special Terms: References

Generic Core Scales:

- Varni JW, et al. The PedsQL™: Measurement Model for the Pediatric Quality of Life Inventory.Medical Care,1999; 37(2):126-139

© Mapi Research Trust. The unauthorized modification and use of any portion of this document is prohibited.

Mapi Research Trust

- Varni, J.W., et al. The PedsQL™ 4.0: Reliability and validity of the Pediatric Quality of Life Inventory™ Version 4.0 Generic Core Scales in healthy and patient populations. Medical Care,2001; 39(8): 800-812.

- Varni, J.W., et al. (2002). The PedsQL™ 4.0 Generic Core Scales: Sensitivity, responsiveness, and impact on clinical decision-making.Journal of Behavioral Medicine, 25, 175-193.

- Varni, J.W., et al. (2003). The PedsQL™ 4.0 as a pediatric population health measure: Feasibility, reliability, and validity.Ambulatory Pediatrics, 3, 329-341.

- Chan, K.S., Mangione-Smith, R., Burwinkle, T.M., Rosen, M., & Varni, J.W. (2005). The PedsQL™: Reliability and validity of the Short-Form Generic Core Scales and Asthma Module.Medical Care, 43, 256-265.

- Varni, J.W., & Limbers, C.A. (2009). The PedsQL™ 4.0 Generic Core Scales Young Adult Version: Feasibility, reliability and validity in a university student population.Journal of Health Psychology, 14, 611-622.

Asthma Module:

- Varni, J.W., Burwinkle, T.M., Rapoff, M.A., Kamps, J.L., & Olson, N. The PedsQL™ in pediatric asthma: Reliability and validity of the Pediatric Quality of Life Inventory™ Generic Core Scales and Asthma Module. Journal of Behavioral Medicine, 2004; 27:297-318.

- Chan, K.S., Mangione-Smith, R., Burwinkle, T.M, Rosen, M., & Varni, J.W. (2005). The PedsQL™: Reliability and validity of the Short-Form Generic Core Scales and Asthma Module. Medical Care, 43, 256-265.

Brain Tumor Module:

- Palmer, S.N., Meeske, K.A., Katz, E.R., Burwinke, T.M., & Varni, J.W. (2007). The PedsQL™ Brain Tumor Module: Initial reliability and validity. Pediatric Blood and Cancer, 49, 287-293.

Cancer Module:

- Varni, J.W., Burwinkle, T.M., Katz, E.R., Meeske, K., & Dickinson, P. The PedsQL™ in pediatric cancer: Reliability and validity of the Pediatric Quality of Life Inventory™ Generic Core Scales, Multidimensional Fatigue Scale, and Cancer Module.Cancer, 2002; 94: 2090-2106.

- Robert RS, Paxton RJ, Palla SL, Yang G, Askins MA, Joy SE, Ater JL. Feasibility, reliability, and validity of the pediatric quality of life inventory™ generic core scales, cancer module, and multidimensional fatigue scale in long-term adult survivors of pediatric cancer.Pediatric Blood & Cancer2012;59:703–707.

Cerebral Palsy Module:

© Mapi Research Trust. The unauthorized modification and use of any portion of this document is prohibited.

- Varni JW, Burwinkle TM, Berrin SJ, Sherman SA, Artavia K, Malcarne VL, Chambers HG (2006). The PedsQL™ in Pediatric Cerebral Palsy: Reliability, Validity, and Sensitivity of the Generic Core Scales and Cerebral Palsy Module.Developmental Medicine and Child Neurology, 48: 442-449.

Cardiac Module:

- Uzark, K., Jones, K., Burwinkle, T.M., & Varni, J.W. The Pediatric Quality of Life Inventory™ in children with heart disease.Progress in Pediatric Cardiology, 2003; 18:141-148.

- Uzark, K., Jones, K., Slusher, J., Limbers, C.A., Burwinkle, T.M., & Varni, J.W. (2008). Quality of life in children with heart disease as perceived by children and parents. Pediatrics, 121, e1060-e1067.

Cognitive Functioning Scale:

- McCarthy, M.L., MacKenzie, E.J., Durbin, D.R., Aitken, M.E., Jaffe, K.M., Paidas, C.N. et al. (2005). The Pediatric Quality of Life Inventory: An evaluation of its reliability and validity for children with traumatic brain injury.Archives of Physical Medicine and Rehabilitation, 86, 1901-1909.

- Varni, J.W., Burwinkle, T.M., Katz, E.R., Meeske, K., & Dickinson, P. (2002). The PedsQL™ in pediatric cancer: Reliability and validity of the Pediatric Quality of Life Inventory Generic Core Scales, Multidimensional Fatigue Scale, and Cancer Module.Cancer, 94, 2090-2106.

- Varni, J.W., Limbers, C.A., Sorensen, L.G., Neighbors, K., Martz, K., Bucuvalas, J.C., & Alonso, E.M. (2011). PedsQL™ Cognitive Functioning Scale in pediatric liver transplant recipients: Feasibility, reliability and validity.Quality of Life Research, 20, 913–921.

Diabetes Module:

- Varni, J.W., Curtis, B.H., Abetz, L.N., Lasch, K.E., Piault, E.C., & Zeytoonjian, A.A. (2013). Content validity of the PedsQL™ 3.2 Diabetes Module in newly diagnosed patients with Type 1 Diabetes Mellitus ages 8-45.Quality of Life Research. 22, 2169–2181.

- Varni, J.W., Burwinkle, T.M., Jacobs, J.R., Gottschalk, M., Kaufman, F., & Jones, K.L. The PedsQL™ in Type 1 and Type 2 diabetes: Reliability and validity of the Pediatric Quality of Life Inventory™ Generic Core Scales and Type 1 Diabetes Module.Diabetes Care, 2003; 26: 631-637.

- Nansel, T.R., Weisberg-Benchell, J., Wysocki, T., Laffel, L. & Anderson, B. (2008). Quality of life in children with Type 1 diabetes: A comparison of general and disease-specific measures and support for a unitary diabetes quality of life construct. Diabetic Medicine, 25, 1316-1323.

- Naughton, M.J., Ruggiero, A.M., Lawrence, J.M., Imperatore, G., Klingensmith, G.J. Waitzfelder, B., McKeown, R.E., Standiford, D.A., Liese, A.D., & Loots, B. (2008). Health-related quality of life of children and adolescents with type 1 or type 2 diabetes mellitus: SEARCH for Diabetes In Youth Study.Archives of Pediatrics and Adolescent Medicine, 162, 649-657.

- Hilliard, M.E., Lawrence, J.M., Modi, A.C., Anderson, A., Crume, T., Dolan, L.M., Merchant, A.T., Yi-Frazier, J.P.,

© Mapi Research Trust. The unauthorized modification and use of any portion of this document is prohibited.

& Hood, K.K. (2013). Identification of minimal clinically important difference scores of the Pediatric Quality of Life Inventory in children, adolescents, and young adults with Type 1 and Type 2 diabetes.Diabetes Care, 36, 1891–1897.

Duchenne Muscular Dystrophy Module:

- Uzark, K., King, E., Cripe, L., Spicer, R., Sage, J., Kinnett, K., Wong, B., Pratt, J., & Varni, J.W. (2012). Health-related quality of life in children and adolescents with Duchenne Muscular Dystrophy.Pediatrics, 130, e1559-e1566.

-

End Stage Renal Disease Module:

- Goldstein, S.L., Graham, N., Warady, B.A., Seikaly, M., McDonald, R., Burwinkle, T.M., Limbers, C.A., & Varni, J.W. (2008). Measuring health-related quality of life in children with ESRD: Performance of the Generic and ESRD-Specific Instrument of the Pediatric Quality of Life Inventory™ (PedsQL™). American Journal of Kidney Diseases, 51, 285-297.

Eosinophilic Esophagitis:

- Franciosi, J.P., Hommel, K.A., Bendo, C.B., King, E.C., Collins, M.H., Eby, M.D., Marsolo, K., Abonia, J.P., von Tiehl, K.F., Putnam, P.E., Greenler, A.J., Greenberg, A.B., Bryson, R.A., Davis, C.M., Olive, A.P., Gupta, S.K., Erwin, E.A., Klinnert, M.D., Spergel, J.M., Denham, J.M., Furuta, G.T., Rothenberg, M.E., & Varni, J.W. (2013). PedsQL™ Eosinophilic Esophagitis Module: Feasibility, reliability and validity.Journal of Pediatric Gastroenterology & Nutrition, 57, 57-66.

- Franciosi, J.P., Hommel, K.A., Greenberg, A.B., Debrosse, C.W., Greenler, A.J., Abonia, J.P., Rothenberg, M.E., & Varni, J.W. (2012). Development of the Pediatric Quality of Life Inventory™ Eosinophilic Esophagitis Module items: Qualitative methods.BMC Gastroenterology, 12:135, 1-8.

- Franciosi J.P., Hommel, K.A., Debrosse, C.W., Greenberg, A.B., Greenler, A.J., Abonia, J.P., Rothenberg, M.E., & Varni, J.W. (2012). Quality of life in paediatric eosinophilic oesophagitis: What is important to patients?Child: Care, Health and Development, 38, 477–483.

Family impact Module:

- Varni, J.W., Sherman, S.A., Burwinkle, T.M., Dickinson, P.E., & Dixon, P. (2004). The PedsQL™ Family Impact Module: Preliminary reliability and validity. Health and Quality of Life Outcomes; 2 (55), 1-6.

- Medrano, G.R., Berlin, K.S., & Davies, W.H. (2013). Utility of the PedsQL™ Family Impact Module: Assessing the psychometric properties in a community sample.Quality of Life Research. 22, 2899-2907.

- Jiang, X., Sun, L., Wang, B., Yang, X., Shang, L., & Zhang, Y. (2013). Health-related quality of life among

© Mapi Research Trust. The unauthorized modification and use of any portion of this document is prohibited.

Mapi Research Trust

children with recurrent respiratory tract infections in Xi'an, China.PLoS One, 8(2): e56945.

- Mano, K.E., Khan, K.A., Ladwig, R.J., & Weisman, S.J. (2011). The impact of pediatric chronic pain on parents' health-related quality of life and family functioning: Reliability and validity of the PedsQL 4.0 Family Impact Module.Journal of Pediatric Psychology, 36, 517-527.

Gastrointestinal Symptoms Module:

- Varni, J.W., Bendo, C.B., Denham, J., Shulman, R.J., Self, M.M., Neigut, D.A., Nurko S., Patel, A.S, Franciosi, J.P., Saps, M., Verga, B., Smith, A., Yeckes, A., Heinz, N., Langseder, A., Saeed, S., Zacur, G.M., & Pohl, J.F. (in press). PedsQL™ Gastrointestinal Symptoms Module: Feasibility, reliability, and validity.Journal of Pediatric Gastroenterology & Nutrition.

- Varni, J.W., Bendo, C.B., Denham, J., Shulman, R.J., Self, M.M., Neigut, D.A., Nurko, S., Patel, A.S, Franciosi, J.P., Saps, M., Yeckes, A., Langseder, A., Saeed, S., & Pohl, J.F. (in press). PedsQL™ Gastrointestinal Symptoms Scales and Gastrointestinal Worry Scales in pediatric patients with functional and organic gastrointestinal diseases in comparison to healthy controls.Quality of Life Research.

- Varni, J.W., Kay, M.T., Limbers, C.A., Franciosi, J.P., & Pohl, J.F. (2012). PedsQL™ Gastrointestinal Symptoms Module item development: Qualitative methods.Journal of Pediatric Gastroenterology & Nutrition, 54, 664-671.

Gastrointestinal Symptoms Scales:

- Varni, J.W., Bendo, C.B., Denham, J., Shulman, R.J., Self, M.M., Neigut, D.A., Nurko S., Patel, A.S, Franciosi, J.P., Saps, M., Verga, B., Smith, A., Yeckes, A., Heinz, N., Langseder, A., Saeed, S., Zacur, G.M., & Pohl, J.F. (2014). PedsQL™ Gastrointestinal Symptoms Module: Feasibility, reliability, and validity. Journal of Pediatric Gastroenterology & Nutrition,59, 347–355.

- Varni, J.W., Bendo, C.B., Denham, J., Shulman, R.J., Self, M.M., Neigut, D.A., Nurko, S., Patel, A.S, Franciosi, J.P., Saps, M., Yeckes, A., Langseder, A., Saeed, S., & Pohl, J.F. (in press). PedsQL™ Gastrointestinal Symptoms Scales and Gastrointestinal Worry Scales in pediatric patients with functional and organic gastrointestinal diseases in comparison to healthy controls.Quality of Life Research.

- Varni, J.W., Kay, M.T., Limbers, C.A., Franciosi, J.P., & Pohl, J.F. (2012). PedsQL™ Gastrointestinal Symptoms Module item development: Qualitative methods.Journal of Pediatric Gastroenterology & Nutrition, 54, 664-671.

General Well-Being Scale:

- Varni, J.W., Seid, M., & Kurtin, P.S. (1999). Pediatric health-related quality of life measurement technology: A guide for health care decision makes. Journal of Clinical Outcomes Management, 6, 33-40.

- Hallstrand, T.S., Curtis, J.R., Aitken, M.L., & Sullivan, S.D. (2003). Quality of life in adolescents with mild asthma. Pediatric Pulmonology, 36, 536-543.

© Mapi Research Trust. The unauthorized modification and use of any portion of this document is prohibited.

Healthcare Satisfaction Generic Module:

- Varni, J.W., Burwinkle, T.M., Dickinson, P., Sherman, S.A., Dixon, P., Ervice, J.A., Leyden, P.A. & Sadler, B.L. (2004). Evaluation of the built environment at a Children's Convalescent Hospital: Development of the Pediatric Quality of Life Inventory™ Parent and Staff Satisfaction Measures for pediatric health care facilities. Journal of Developmental and Behavioral Pediatrics, 2004; 25:10-25.

- Li, J., Yuan, L., Wu, Y., Luan, Y., & Hao, Y. (2013). The Chinese version of the Pediatric Quality of Life Inventory™ (PedsQL™) healthcare satisfaction generic module (version 3.0): Psychometric evaluation. Health and Quality of Life Outcomes, 11(1):113.

- de Souza, F.M., Molina, J., Terreri, M.T., Hilário, M.O., & Len, CA. (2012). Reliability of the Pediatric Quality of Life Inventory - Healthcare Satisfaction Generic Module 3.0 version for the assessment of the quality of care of children with chronic diseases. Journal of Pediatrics (Rio J), 88, 54-60.

Health Care Satisfaction Module specific for Hematology/Oncology:

- Varni, J.W., Quiggins, D.J.L., & Ayala, G.X. (2000). Development of the Pediatric Hematology/Oncology Parent Satisfaction survey. Children's Health Care, 29, 243-255.

Infant Scales:

- Varni, J.W., Limbers, C.A., Neighbors, K., Schulz, K., Lieu, J.E.C., Heffer, R.W., Tuzinkiewicz, K., Mangione-Smith, R., Zimmerman, J.J., & Alonso, E.M. (2011). The PedsQL™ Infant Scales: Feasibility, internal consistency reliability and validity in healthy and ill infants.Quality of Life Research, 20, 45-55.

- Grindler, D.J., Blank, S.J., Schulz, K.A., Witsell, D.L., & Lieu, J.E. (2014). Impact of otitis media severity on children's quality of life.Otolaryngology-Head and Neck Surgery, 151, 333–340.

- Bell, N., Kruse, S., Simons, R.K., & Brussoni, M. (2014). A spatial analysis of functional outcomes and quality of life outcomes after pediatric injury.Injury Epidemiology, 1:16, 1-10.

Multidimensional Fatigue Scale:

- Varni, J.W., Burwinkle, T.M., Katz, E.R., Meeske, K., & Dickinson, P. (2002). The PedsQL™ in pediatric cancer: Reliability and validity of the Pediatric Quality of Life Inventory™ Generic Core Scales, Multidimensional Fatigue Scale, and Cancer Module.Cancer, 94, 2090-2106.

- Varni, J. W., Beaujean, A., & Limbers, C. A. (2013). Factorial invariance of pediatric patient self-reported fatigue across age and gender: A multigroup confirmatory factor analysis approach utilizing the PedsQL™ Multidimensional Fatigue Scale.Quality of Life Research, 22, 2581-2594.

- Varni, J.W., Burwinkle, T.M., & Szer, I.S. (2004). The PedsQL™ Multidimensional Fatigue Scale in

© Mapi Research Trust. The unauthorized modification and use of any portion of this document is prohibited.

pediatric rheumatology: Reliability and validity. Journal of Rheumatology; 31, 2494-2500.

- Varni, J.W., & Limbers, C.A. (2008). The PedsQL™ Multidimensional Fatigue Scale in young adults: Feasibility, reliability and validity in a university student population. Quality of Life Research, 17, 105-114.

- Panepinto, J.A., Torres, S., Bendo, C.B., McCavit, T.L., Dinu, B., Sherman-Bien, S., Bemrich-Stolz, C., & Varni, J.W. (2014). PedsQL™ Multidimensional Fatigue Scale in sickle cell disease: Feasibility, reliability and validity.Pediatric Blood & Cancer, 61, 171–177.

Neurofibromatosis Type 1 Module:

- Nutakki, K., Hingtgen, C.M., Monahan, P., Varni, J.W., & Swigonski, N.L. (2013). Development of the adult PedsQL™ Neurofibromatosis Type 1 Module: Initial feasibility, reliability and validity.Health and Quality of Life Outcomes, 11:21, 1-9

Neuromuscular Module:

- Iannaccone, S.T., Hynan, L.S., Morton, A., Buchanan, R., Limbers, C.A., & Varni, J.W. (2009). The PedsQL™ in pediatric patients with Spinal Muscular Atrophy: Feasibility, reliability, and validity of the Pediatric Quality of Life Inventory™ Generic Core Scales and Neuromuscular Module.Neuromuscular Disorders, 19, 805-812.

- Davis, S.E., Hynan, L.S., Limbers, C.A., Andersen, C.M., Greene, M.C., Varni, J.W., & Iannaccone, S.T. (2010). The PedsQL™ in pediatric patients with Duchenne Muscular Dystrophy: Feasibility, reliability, and validity of the Pediatric Quality of Life Inventory™ Neuromuscular Module and Generic Core Scales.Journal of Clinical Neuromuscular Disease, 11, 97-109.

Oral Health Scale:

- Steele, M.M., Steele, R.G., & Varni, J.W. (2009). Reliability and validity of the PedsQL™ Oral Health Scale: Measuring the relationship between child oral health and health-related quality of life.Children's Health Care, 38, 228-224.

Pediatric Pain Coping Inventory™:

- Varni, J.W., Waldron, S.A., Gragg, R.A., Rapoff, M.A., Bernstein, B.H., Lindsley, C.B., & Newcomb, M.D (1996). Development of the Waldron/Varni Pediatric Pain Coping Inventory. Pain, 67, 141-150.

Pediatric Pain Questionnaire:

- Varni, J.W., Thompson, K.L., & Hanson, V. (1987). The Varni/Thompson Pediatric Pain Questionnaire: I. Chronic musculoskeletal pain in juvenile rheumatoid arthritis.Pain, 28, 27-38.

© Mapi Research Trust. The unauthorized modification and use of any portion of this document is prohibited.

Present Functioning Visual Analogue Scales:

- Sherman, S.A., Eisen, S., Burwinkle, T.M., & Varni, J.W. (2006). The PedsQL™ Present Functioning Visual Analogue Scales: Preliminary reliability and validity.Health and Quality of Life Outcomes, 4:75, 1-10.

Sickle Cell Disease Module:

- Panepinto, J.A., Torres, S., Bendo, C.B., McCavit, T.L., Dinu, B., Sherman-Bien, S., Bemrich-Stolz, C., & Varni, J.W. (2013). PedsQL™ Sickle Cell Disease Module: Feasibility, reliability and validity.Pediatric Blood & Cancer, 60, 1338–1344.

- Panepinto, J.A., Torres, S., & Varni. J.W. (2012). Development of the PedsQL™ Sickle Cell Disease Module items: Qualitative methods.Quality of Life Research, 21, 341-357.

Stem Cell Transplant Module:

- Lawitschka, A., Güclü, E.D., Varni, J.W., Putz, M., Wolff, D., Pavletic, S., Greinix, H., Peters, C., & Felder-Puig, R. (2014). Health-related quality of life in pediatric patients after allogeneic SCT: Development of the PedsQL™ Stem Cell Transplant Module and results of a pilot study. Bone Marrow Transplantation, 49, 1093–1097.

Rheumatology Module:

- Varni, J.W., Seid, M., Knight, T.S., Burwinkle, T.M., Brown, J., & Szer, I.S. (2002). The PedsQL™ in pediatric rheumatology: Reliability, validity, and responsiveness of the Pediatric Quality of Life Inventory™ Generic Core Scales and Rheumatology Module.Arthritis and Rheumatism, 2002; 46: 714-725.

Transplant Module:

- Weissberg-Benchell, J., Zielinski, T.E., Rodgers, S., Greenley, R.N., Askenazi, D., Goldstein, S.L., Fredericks, E.M., McDiarmid, S., Williams, L., Limbers, C.A., Tuzinkiewicz, K., Lerret, S., Alonso, E.M., & Varni, J.W. (2010). Pediatric health-related quality of life: Feasibility, reliability and validity of the PedsQL™ Transplant Module.American Journal of Transplantation, 10, 1677-1685.

© Mapi Research Trust. The unauthorized modification and use of any portion of this document is prohibited.

N° de identificação: ____________

Data: ____________________

PedsQL™

Questionário pediátrico sobre qualidade de vida

Versão 4.0 – Português (Brasil)

RELATO DO/A ADOLESCENTE (13 a 18 anos)

INSTRUÇÕES

A próxima página contém uma lista de coisas com as quais você pode ter dificuldade.
Por favor, conte-nos se você **tem tido dificuldade** com cada uma dessas coisas durante o **ÚLTIMO MÊS**, fazendo um "X" no número:

0 se você **nunca** tem dificuldade com isso
1 se você **quase nunca** tem dificuldade com isso
2 se você **algumas vezes** tem dificuldade com isso
3 se você **muitas vezes** tem dificuldade com isso
4 se você **quase sempre** tem dificuldade com isso

Não existem respostas certas ou erradas.
Caso você não entenda alguma pergunta, por favor, peça ajuda.

Copyright © 1998 JW Varni, Ph.D. Todos os direitos reservados.
Não pode ser reproduzido sem autorização prévia.

*Durante o **ÚLTIMO MÊS**, você tem tido **dificuldade** com cada uma das coisas abaixo?*

**SOBRE MINHA SAÚDE E MINHAS ATIVIDADES** *(dificuldade para...)*	Nunca	Quase nunca	Algumas vezes	Muitas vezes	Quase sempre
1. Para mim é difícil andar mais de um quarteirão	0	1	2	3	4
2. Para mim é difícil correr	0	1	2	3	4
3. Para mim é difícil praticar esportes ou fazer exercícios físicos	0	1	2	3	4
4. Para mim é difícil levantar coisas pesadas	0	1	2	3	4
5. Para mim é difícil tomar banho de banheira ou de chuveiro sozinho/a	0	1	2	3	4
6. Para mim é difícil ajudar nas tarefas domésticas	0	1	2	3	4
7. Eu sinto dor	0	1	2	3	4
8. Eu tenho pouca energia ou disposição	0	1	2	3	4

**SOBRE MEUS SENTIMENTOS** *(dificuldade para...)*	Nunca	Quase nunca	Algumas vezes	Muitas vezes	Quase sempre
1. Eu sinto medo	0	1	2	3	4
2. Eu me sinto triste	0	1	2	3	4
3. Eu sinto raiva	0	1	2	3	4
4. Eu durmo mal	0	1	2	3	4
5. Eu me preocupo com o que vai acontecer comigo	0	1	2	3	4

**COMO EU CONVIVO COM OUTRAS PESSOAS** *(dificuldades para...)*	Nunca	Quase nunca	Algumas vezes	Muitas vezes	Quase sempre
1. Eu tenho dificuldade para conviver com outros / outras adolescentes	0	1	2	3	4
2. Os outros / as outras adolescentes não querem ser meus amigos / minhas amigas	0	1	2	3	4
3. Os outros / as outras adolescentes implicam comigo	0	1	2	3	4
4. Eu não consigo fazer coisas que outros / outras adolescentes da minha idade fazem	0	1	2	3	4
5. Para mim é difícil acompanhar os / as adolescentes da minha idade	0	1	2	3	4

**SOBRE A ESCOLA** *(dificuldades para...)*	Nunca	Quase nunca	Algumas vezes	Muitas vezes	Quase sempre
1. É difícil prestar atenção na aula	0	1	2	3	4
2. Eu esqueço as coisas	0	1	2	3	4
3. Eu tenho dificuldade para acompanhar a minha turma nas tarefas escolares	0	1	2	3	4
4. Eu falto à aula por não estar me sentindo bem	0	1	2	3	4
5. Eu falto à aula para ir ao médico ou ao hospital	0	1	2	3	4

Copyright © 1998 JW Varni, Ph.D. Todos os direitos reservados.
Não pode ser reproduzido sem autorização prévia.

N° de identificação: ____________

Data: ______________________

PedsQL™

Questionário pediátrico sobre qualidade de vida

Versão 4.0 – Português (Brasil)

RELATO DA CRIANÇA (8 a 12 anos)

INSTRUÇÕES

A próxima página contém uma lista de coisas com as quais você pode ter dificuldade.
Por favor, conte-nos se você **tem tido dificuldade** com cada uma dessas coisas durante o **ÚLTIMO MÊS**, fazendo um "X" no número:

0 se você **nunca** tem dificuldade com isso
1 se você **quase nunca** tem dificuldade com isso
2 se você **algumas vezes** tem dificuldade com isso
3 se você **muitas vezes** tem dificuldade com isso
4 se você **quase sempre** tem dificuldade com isso

Não existem respostas certas ou erradas.
Caso você não entenda alguma pergunta, por favor, peça ajuda.

Copyright © 1998 JW Varni, Ph.D. Todos os direitos reservados.
Não pode ser reproduzido sem autorização prévia.

*Durante o ÚLTIMO MÊS, você tem tido **dificuldade** com cada uma das coisas abaixo?*

**SOBRE MINHA SAÚDE E MINHAS ATIVIDADES** *(dificuldade para...)*	Nunca	Quase nunca	Algumas vezes	Muitas vezes	Quase sempre
1. Para mim é difícil andar mais de um quarteirão	0	1	2	3	4
2. Para mim é difícil correr	0	1	2	3	4
3. Para mim é difícil praticar esportes ou fazer exercícios físicos	0	1	2	3	4
4. Para mim é difícil levantar coisas pesadas	0	1	2	3	4
5. Para mim é difícil tomar banho de banheira ou de chuveiro sozinho/a	0	1	2	3	4
6. Para mim é difícil ajudar nas tarefas domésticas	0	1	2	3	4
7. Eu sinto dor	0	1	2	3	4
8. Eu me sinto cansado/a	0	1	2	3	4

**SOBRE MEUS SENTIMENTOS** *(dificuldade para...)*	Nunca	Quase nunca	Algumas vezes	Muitas vezes	Quase sempre
1. Eu sinto medo	0	1	2	3	4
2. Eu me sinto triste	0	1	2	3	4
3. Eu sinto raiva	0	1	2	3	4
4. Eu durmo mal	0	1	2	3	4
5. Eu me preocupo com o que vai acontecer comigo	0	1	2	3	4

**COMO EU CONVIVO COM OUTRAS PESSOAS** *(dificuldades para...)*	Nunca	Quase nunca	Algumas vezes	Muitas vezes	Quase sempre
1. Eu tenho dificuldade para conviver com outras crianças	0	1	2	3	4
2. As outras crianças não querem ser minhas amigas	0	1	2	3	4
3. As outras crianças implicam comigo	0	1	2	3	4
4. Eu não consigo fazer coisas que outras crianças da minha idade fazem	0	1	2	3	4
5. Para mim é difícil acompanhar a brincadeira com outras crianças	0	1	2	3	4

**SOBRE A ESCOLA** *(dificuldades para...)*	Nunca	Quase nunca	Algumas vezes	Muitas vezes	Quase sempre
1. É difícil prestar atenção na aula	0	1	2	3	4
2. Eu esqueço as coisas	0	1	2	3	4
3. Eu tenho dificuldade para acompanhar a minha turma nas tarefas escolares	0	1	2	3	4
4. Eu falto à aula por não estar me sentindo bem	0	1	2	3	4
5. Eu falto à aula para ir ao médico ou ao hospital	0	1	2	3	4

Copyright © 1998 JW Varni, Ph.D. Todos os direitos reservados.
Não pode ser reproduzido sem autorização prévia.

SPIRIT 2013 Checklist: Recommended items to address in a clinical trial protocol and related documents*

Section/item	Item No	Description	Addressed on page number
**Administrative information**			
Title	1	Descriptive title identifying the study design, population, interventions, and, if applicable, trial acronym	______1______
Trial registration	2a	Trial identifier and registry name. If not yet registered, name of intended registry	______1______
	2b	All items from the World Health Organization Trial Registration Data Set	______5______
Protocol version	3	Date and version identifier	______1______
Funding	4	Sources and types of financial, material, and other support	______1______
Roles and responsibilities	5a	Names, affiliations, and roles of protocol contributors	______1______
	5b	Name and contact information for the trial sponsor	_____N/A_____
	5c	Role of study sponsor and funders, if any, in study design; collection, management, analysis, and interpretation of data; writing of the report; and the decision to submit the report for publication, including whether they will have ultimate authority over any of these activities	_____N/A_____
	5d	Composition, roles, and responsibilities of the coordinating centre, steering committee, endpoint adjudication committee, data management team, and other individuals or groups overseeing the trial, if applicable (see Item 21a for data monitoring committee)	_____N/A_____

Introduction

Background and rationale	6a	Description of research question and justification for undertaking the trial, including summary of relevant studies (published and unpublished) examining benefits and harms for each intervention	____3,4,5____
	6b	Explanation for choice of comparators	______4,5____
Objectives	7	Specific objectives or hypotheses	______5_____
Trial design	8	Description of trial design including type of trial (eg, parallel group, crossover, factorial, single group), allocation ratio, and framework (eg, superiority, equivalence, noninferiority, exploratory)	______5_____

Methods: Participants, interventions, and outcomes

Study setting	9	Description of study settings (eg, community clinic, academic hospital) and list of countries where data will be collected. Reference to where list of study sites can be obtained	_______5____
Eligibility criteria	10	Inclusion and exclusion criteria for participants. If applicable, eligibility criteria for study centres and individuals who will perform the interventions (eg, surgeons, psychotherapists)	__6, table 1___
Interventions	11a	Interventions for each group with sufficient detail to allow replication, including how and when they will be administered	_______6,7___
	11b	Criteria for discontinuing or modifying allocated interventions for a given trial participant (eg, drug dose change in response to harms, participant request, or improving/worsening disease)	______7______
	11c	Strategies to improve adherence to intervention protocols, and any procedures for monitoring adherence (eg, drug tablet return, laboratory tests)	______7______
	11d	Relevant concomitant care and interventions that are permitted or prohibited during the trial	______6,7____
Outcomes	12	Primary, secondary, and other outcomes, including the specific measurement variable (eg, systolic blood pressure), analysis metric (eg, change from baseline, final value, time to event), method of aggregation (eg, median, proportion), and time point for each outcome. Explanation of the clinical relevance of chosen efficacy and harm outcomes is strongly recommended	______8______
Participant timeline	13	Time schedule of enrolment, interventions (including any run-ins and washouts), assessments, and visits for participants. A schematic diagram is highly recommended (see Figure)	_6,7,8,Figure 3_

Sample size	14	Estimated number of participants needed to achieve study objectives and how it was determined, including clinical and statistical assumptions supporting any sample size calculations	_______6____
Recruitment	15	Strategies for achieving adequate participant enrolment to reach target sample size	_______6,7___

Methods: Assignment of interventions (for controlled trials)

Allocation:			
Sequence generation	16a	Method of generating the allocation sequence (eg, computer-generated random numbers), and list of any factors for stratification. To reduce predictability of a random sequence, details of any planned restriction (eg, blocking) should be provided in a separate document that is unavailable to those who enrol participants or assign interventions	____N/A______
Allocation concealment mechanism	16b	Mechanism of implementing the allocation sequence (eg, central telephone; sequentially numbered, opaque, sealed envelopes), describing any steps to conceal the sequence until interventions are assigned	____N/A______
Implementation	16c	Who will generate the allocation sequence, who will enrol participants, and who will assign participants to interventions	____N/A______
Blinding (masking)	17a	Who will be blinded after assignment to interventions (eg, trial participants, care providers, outcome assessors, data analysts), and how	____N/A______
	17b	If blinded, circumstances under which unblinding is permissible, and procedure for revealing a participant's allocated intervention during the trial	____N/A______

Methods: Data collection, management, and analysis

Data collection methods	18a	Plans for assessment and collection of outcome, baseline, and other trial data, including any related processes to promote data quality (eg, duplicate measurements, training of assessors) and a description of study instruments (eg, questionnaires, laboratory tests) along with their reliability and validity, if known. Reference to where data collection forms can be found, if not in the protocol	_____6,7,8____
	18b	Plans to promote participant retention and complete follow-up, including list of any outcome data to be collected for participants who discontinue or deviate from intervention protocols	_____7_______

Data management	19	Plans for data entry, coding, security, and storage, including any related processes to promote data quality (eg, double data entry; range checks for data values). Reference to where details of data management procedures can be found, if not in the protocol	_____8______
Statistical methods	20a	Statistical methods for analysing primary and secondary outcomes. Reference to where other details of the statistical analysis plan can be found, if not in the protocol	______8,9____
	20b	Methods for any additional analyses (eg, subgroup and adjusted analyses)	______8,9____
	20c	Definition of analysis population relating to protocol non-adherence (eg, as randomised analysis), and any statistical methods to handle missing data (eg, multiple imputation)	______N/A______
**Methods: Monitoring**			
Data monitoring	21a	Composition of data monitoring committee (DMC); summary of its role and reporting structure; statement of whether it is independent from the sponsor and competing interests; and reference to where further details about its charter can be found, if not in the protocol. Alternatively, an explanation of why a DMC is not needed	______N/A______
	21b	Description of any interim analyses and stopping guidelines, including who will have access to these interim results and make the final decision to terminate the trial	_______N/A_____
Harms	22	Plans for collecting, assessing, reporting, and managing solicited and spontaneously reported adverse events and other unintended effects of trial interventions or trial conduct	_______N/A_____
Auditing	23	Frequency and procedures for auditing trial conduct, if any, and whether the process will be independent from investigators and the sponsor	_______N/A_____
**Ethics and dissemination**			
Research ethics approval	24	Plans for seeking research ethics committee/institutional review board (REC/IRB) approval	_______5______
Protocol amendments	25	Plans for communicating important protocol modifications (eg, changes to eligibility criteria, outcomes, analyses) to relevant parties (eg, investigators, REC/IRBs, trial participants, trial registries, journals, regulators)	_______9______

Consent or assent	26a	Who will obtain informed consent or assent from potential trial participants or authorised surrogates, and how (see Item 32)	________5_____
	26b	Additional consent provisions for collection and use of participant data and biological specimens in ancillary studies, if applicable	________5_____
Confidentiality	27	How personal information about potential and enrolled participants will be collected, shared, and maintained in order to protect confidentiality before, during, and after the trial	________5_____
Declaration of interests	28	Financial and other competing interests for principal investigators for the overall trial and each study site	________1_____
Access to data	29	Statement of who will have access to the final trial dataset, and disclosure of contractual agreements that limit such access for investigators	________9_____
Ancillary and post-trial care	30	Provisions, if any, for ancillary and post-trial care, and for compensation to those who suffer harm from trial participation	_______N/A_____
Dissemination policy	31a	Plans for investigators and sponsor to communicate trial results to participants, healthcare professionals, the public, and other relevant groups (eg, via publication, reporting in results databases, or other data sharing arrangements), including any publication restrictions	_______9______
	31b	Authorship eligibility guidelines and any intended use of professional writers	_______1,9______
	31c	Plans, if any, for granting public access to the full protocol, participant-level dataset, and statistical code	_______9_______
**Appendices**			
Informed consent materials	32	Model consent form and other related documentation given to participants and authorised surrogates	__supplemental digital content 2 and 3______
Biological specimens	33	Plans for collection, laboratory evaluation, and storage of biological specimens for genetic or molecular analysis in the current trial and for future use in ancillary studies, if applicable	___N/A_______

*It is strongly recommended that this checklist be read in conjunction with the SPIRIT 2013 Explanation & Elaboration for important clarification on the items. Amendments to the protocol should be tracked and dated. The SPIRIT checklist is copyrighted by the SPIRIT Group under the Creative Commons "Attribution-NonCommercial-NoDerivs 3.0 Unported" license.
